# Brazilian Fetal Cardiology Guidelines - 2019

**DOI:** 10.5935/abc.20190075

**Published:** 2019-05

**Authors:** Simone R. F. Fontes Pedra, Paulo Zielinsky, Cristiane Nogueira Binotto, Cristiane Nunes Martins, Eduardo Sérgio Valério Borges da Fonseca, Isabel Cristina Britto Guimarães, Izabele Vian da Silveira Corrêa, Karla Luiza Matos Pedrosa, Lilian Maria Lopes, Luiz Henrique Soares Nicoloso, Marcia Ferreira Alves Barberato, Marina Maccagnano Zamith

**Affiliations:** 1 Instituto Dante Pazzanese de Cardiologia, São Paulo, SP - Brazil; 2 Hospital do Coração (HCor), São Paulo, SP - Brazil; 3 Instituto de Cardiologia do Rio Grande do Sul, Porto Alegre, RS - Brazil; 4 Hospital Pequeno Príncipe, Curitiba, PR - Brazil; 5 Universidade Positivo, Curitiba, PR - Brazil; 6 Biocor Instituto, Nova Lima, MG - Brazil; 7 Universidade Federal da Paraíba (UFPB), João Pessoa, PB - Brazil; 8 Universidade Federal da Bahia (UFBA), Salvador, BA - Brazil; 9 Hospital Ana Nery, Salvador, BA - Brazil; 10 Ecokidgrafia Serviços Médicos, São Paulo, SP - Brazil; 11 Cardioeco Centro de Diagnóstico Cardiovascular, Curitiba, PR - Brazil; 12 Universidade Federal de São Paulo (UNIFESP), São Paulo, SP - Brazil

**Table:** 

Declaration of potential conflict of interest of authors/collaborators of the Brazilian Fetal Cardiology Guidelines 2019							
If the last three years the author/developer of the Guidelines:							
**Names Members of the Policy**	**Participated in clinical studies and/or experimental trials supported by pharmaceutical or equipment related to the guideline in question**	**Has spoken at events or activities sponsored by industry related to the guideline in question**	**It was (is) advisory board member or director of a pharmaceutical or equipment**	**Committees participated in completion of research sponsored by industry**	**Personal or institutional aid received from industry**	**Produced scientific papers in journals sponsored by industry**	**It shares the industry**
Cristiane Nogueira Binotto	No	No	No	No	No	No	No
Cristiane Nunes Martins	No	No	No	No	No	No	No
Eduardo Srgio Valrio Borges da Fonseca	No	No	No	No	No	No	No
Isabel Cristina Britto Guimare	No	No	No	No	No	No	No
Izabele Vian da Silveira Corra	No	No	No	No	No	No	No
Karla Luiza Matos Pedrosa	No	No	No	No	No	No	No
Lilian Maria Lopes	No	No	No	No	No	No	No
Luiz Henrique Soares Nicoloso	No	No	No	No	No	No	No
Marcia Ferreira Alves Barberato	No	No	No	No	No	No	No
Marina Maccagnano Zamith	No	No	No	No	No	No	No
Paulo Zielinsky	No	No	No	No	No	No	No
Simone R. F. Fontes Pedra	No	No	No	No	No	No	No

## 1. Introduction

Over the years, Fetal Cardiology have been incorporated into the daily practice of Pediatric Cardiology. What was once restricted to a few fetal heart researchers, has slowly been incorporated into health institutions that deal with congenital heart diseases (CHD). Fetal echocardiography has generated extensive knowledge of the natural and modified history of heart diseases in utero, and normal fetal heart physiology and anatomy. The benefits of fetal diagnosis have become unquestionable over the years. Pioneers in the area succeeded in demystifying the fetal heart examination and proving the importance of screening for cardiac abnormalities during obstetric examinations. Prenatal detection rates have increased, and interest in fetal echocardiography is, thus, no longer merely a diagnostic tool; it has gone on to become a tool of the utmost importance in assisting medical and, progressively, interventional treatment of specific anomalies that occur in fetal life.

A vast body of literature currently supports the practice of Fetal Cardiology. In addition to diagnosis, anatomical and functional particularities may be identified in utero, with implications on the delivery planning and pre and postnatal management. Prenatal diagnosis has certainly led to increase the number of babies with complex heart diseases in Pediatric Cardiology hospital beds. Prior to this, children with complex heart diseases did not survive the immediate neonatal period and died in neonatal intensive care units without being diagnosed. Nowadays, these children require increasingly careful and specific management involving Pediatric Cardiology and thus modifying the practice of Neonatal Cardiology.

Despite the vast literature pertinent to Fetal Cardiology, due to the restricted number of cases, there is a lack of studies with large populations and randomization processes, being the information based on observational studies and description of small samples or cases reports. However, the accumulated knowledge is already enough to develop scientific statements or guidelines.

In April 2014, the American Heart Association (AHA) published the first scientific statement for Fetal Cardiology, encompassing all the practical aspects involved in this area, including screening, diagnosis, medical or interventional therapy, counseling, delivery planning, and neonatal treatment. Considering this extremely thorough and highly useful document, we have accepted the challenge of bringing together professionals dedicated to Fetal Cardiology from different regions of Brazil in order to jointly establish guidelines which are adapted to our reality and which also take into consideration knowledge created in Brazil. We believe that the information brought together in this document will be of great use to professionals who face the challenge of dealing with possible abnormalities that affect the fetal heart in their daily practice.

## 2. Screening and Diagnosis of Fetal Heart Disease

### 2.1. Introduction

One of the main aims of prenatal diagnosis is the detection of severe CHD, whose diagnoses, in most cases, depend on delivery planning in a specialized referral center.^1-3^ Although fetal echocardiography, which is traditionally designated for high-risk pregnancies, is quite accurate, the majority of newborns affected by heart diseases in most parts of the world, continue to be born without having been diagnosed. This occur because many cases of CHD affect low-risk groups and are not detected by screening prenatal ultrasound.^4,5^

The concept of prenatal screening for CHD was first suggested in 1985, with the recommendation of incorporating the four-chamber view into routine obstetric ultrasound.^6^ For more than 25 years, countries such as France, the United Kingdom and Spain have recommended examination of the fetal heart during the routine obstetric ultrasound. Nonetheless, after many years of investment in educational training programs, regional variation in detection rates of prenatal heart diseases continue to be high. The classic study by Garne et al.,^7^ conducted in 20 European centers showed that the global detection rate of fetal heart diseases was rather low (25%), France being the country with the best performance (48%), followed by Spain (45%), Germany (40%), and the United Kingdom (35%). Many studies have shown that detection rates of prenatal heart diseases significantly improve with the expansion of scanning planes for cardiac analysis, but they remain well below 50% and continue to lag behind in relation to prenatal detection of other forms of congenital malformation.^8,9^

Faced with this situation, some have argued that fetal echocardiography should be indicated for all pregnancies, given that, in experienced hands, it is able to detect nearly 100% of all cardiac anomalies in fetal life and is considered the gold standard for fetal cardiac diagnosis.^10-13^

Although it is almost intuitive that prenatal detection of heart diseases would improve perinatal results, it has not been easy to prove this observation scientifically, owing to the difficulty of comparing groups with pre- and postnatal diagnoses, which present rather peculiar and discrepant characteristics. The group with prenatal diagnosis often presents with fetal death or early neonatal death before surgery, as it pertains to the much more severe spectrum of fetal cardiac abnormalities, due to the inability of obstetric ultrasound to screen simpler heart diseases, thus resulting in higher global mortality. On the other hand, the group with postnatal diagnosis, that survives the fetal and early neonatal periods until the baby arrives in a tertiary center, has already demonstrated some constitutional advantages for survival.^2^

A study conducted in France comparing perinatal outcome between babies with transposition of the great arteries, with and without prenatal diagnosis, showed, for the first time, that prenatal diagnosis significantly decreased pre- and postoperative mortality.^14^ Other studies have suggested better results for hypoplastic left heart syndrome (HLHS) and coarctation of the aorta when they are diagnosed during fetal life.^15,16^

Efforts and resources should be directed to teaching and training for prenatal screening of CHD by obstetric ultrasound to achieve a better and more uniform pattern of detection, since performing fetal echocardiography in all pregnancies is unrealistic and has yet to be adopted as a health policy in developed countries.^11,13,17^

Table 2.1 shows the main risk factors for fetal heart diseases, divided into absolute risk of ≥ 2% and < 2%.

**Tabela 2.1:** Clinical conditions that increase the risk of fetal heart disease and are formal indications to perform fetal echocardiogram

Absolute risk ≥ 2%		GOR/LOE
Pregestational maternal diabetes mellitus		I/A
Maternal diabetes mellitus diagnosed during the first trimester		I/A
Poorly controlled maternal phenylketonuria,		I/A
Maternal anti-RO and anti-LA (SSA/SSB) antibodies		IIa/B
Maternal medication exposures	ACE	IIa/B
	Retinoic acids	I/B
	Nonsteroidal anti-inflammatory medications during the third trimester	I/A
Maternal rubella during the first semester		I/C
Maternal infection, with fetal myocarditis suspected		I/C
Use of assisted reproduction technology		IIa/A
CHD in first-degree relative (mother, father, or sibling)		I/B
Mendelian inheritance associated with CHD in first- or second-degree relative		I/C
Suspected CHD on obstetric ultrasound		I/B
Suspected noncardiac abnormality on obstetric ultrasound		I/B
Abnormal fetal karyotype		I/C
Fetal bradycardia, tachycardia, or irregular cardiac rhythm		I/C
Increased nuchal translucency > 95% (≥ 3 mm)		IIa/A
Monochorionic twins		I/A
Fetal hydrops or pleural effusion		I/B
**Absolute risk between 1 and 2%**		
Maternal medication exposures	Anticonvulsants	IIb/A
	Lithium	IIb/B
	Vitamin A	IIb/B
	Selected serotonin reuptake inhibitor (only paroxetine)	IIb/A
	Nonsteroidal anti-inflammatory drugs during the first and second trimesters	IIb/B
CHD in second-degree relatives		IIb/B
Fetal abnormality of umbilical cord or placenta		IIb/C
Intra-abdominal fetal venous anomaly		IIb/C
**Absolute risk ≤ 1%**		
Gestational maternal diabetes mellitus with HbA1c < 6%		III/B
Maternal medication exposures	Selected serotonin reuptake inhibitor (excepting paroxetine)	III/A
	Vitamin K antagonists (warfarin)	III/B
Maternal infection other than rubella with seroconversion only		III/C
Isolated CHD in a distant relative (not first- or second-degree)		III/B

ACE: angiotensin-converting enzyme; CHD: congenital heart disease; GOR: grade of recommendation; HbA1c: hemoglobin A1c; LOE: level of evidence.

Source: Adapted from Donofrio et al.^17^

### 2.2. Fetal Heart Screening During Morphological Ultrasound

Considering all these characteristics, we propose a very simple methodology for evaluating the fetal heart, which has been applied in various countries throughout the world. The main advantage of this systematized heart evaluation is that it eliminates the need for complex views and images, avoiding more difficult maneuvers, which is time-consuming and discourage the examiner who neglects this important part of the morphological exam.

**Figure 2.1:**
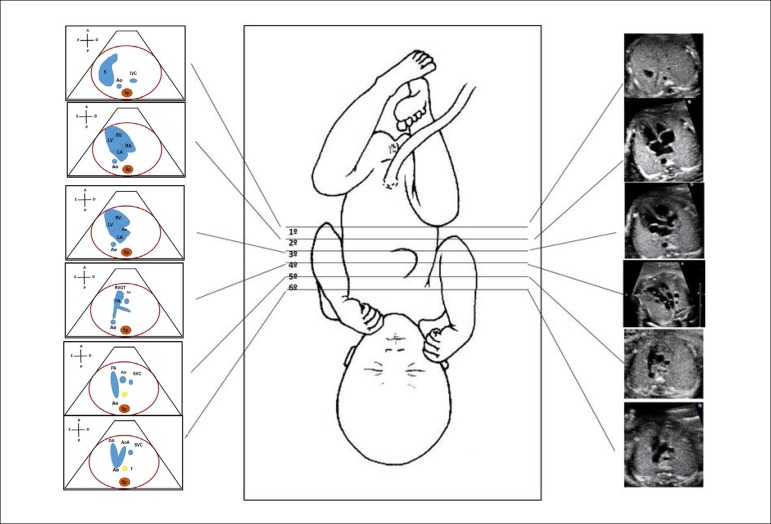
Standardization of fetal heart screening, scanning the fetal vessels and heart from the infradiaphragmatic region towards the cranium. There are 6 levels, being the first exactly below the diaphragm, which allows the identification of the descending aorta and inferior vena cava; second, the four-chamber view; third, left ventricular outflow tract; fourth, right ventricular outflow tract; fifth, three vessel view, and, sixth, three vessel and trachea view. Ao: Aorta; AoA: aortic arch; Asc: ascending; DA: ductus arteriosus; IVC: inferior vena cava; LA: left atrium; LV: left ventricle; PA: pulmonary artery; RA: right atrium; RV: right ventricle; RVOT: right ventricular outflow tract; S: stomach; Sp: spine; SVC: superior vena cava; T: trachea.

With this technique, the fetal heart is evaluated on transverse plane images of the baby only, with no need to rotate the transducer. It starts from the fetal abdomen, from the infradiaphragmatic region to the upper mediastinum, obtaining 6 planes, as shown in Figure 2.1.

#### 2.2.1. Step 1 - 1^st^ Level: Evaluation of the Abdomen to Identify the Abdominal Aorta and the Inferior Vena Cava

This is a transverse view of the fetal abdomen, in the subdiaphragmatic region, and helps to determine the abdominal situs. Before starting, it is necessary to identify the right and left sides of the fetus, according to the fetal presentation; the stomach should be on the fetal left side and the liver on the right. Furthermore, the descending aorta should be seen posterior and to the left, close to the spine, and the inferior vena cava anterior and to the right, within the hepatic parenchyma.

#### 2.2.2. Step 2 - 2^nd^ Level: Four Chamber View

This view is obtained with a transverse scan of the fetal thorax, immediately above the diaphragm. The heart should occupy one third of the thorax, the greater part being in the left hemithorax, with the apex turned to the left. The interventricular septum should be at an angle of approximately 45^th^ with the midline.

The first step for fetal cardiac analysis is the identification of the spine. Opposite to the spine is the anterior wall of the thorax, or sternum. Below is the right ventricle, which is characterized by the moderator band and the tricuspid valve, located a few millimeters displaced to the apex. Returning to the spine, the descending aorta is seen anteriorly as a circle in the mediastinum and, in front of it, is the left atrium. The left atrium is close to the descending aorta and can be identified by the characteristic movement of the foramen ovale flap. Other intracardiac structures, such as the right atrium and the left ventricle, may then be analyzed. They should have dimensions similar to those of the contralateral chambers. The atrioventricular valves should be analyzed in relation to their movement and size of their valve annulus.

In summary, the analysis of the four-chamber view should include the following reference points:

- Spine.
- Descending aorta in a transverse plane.
- Left atrium close to the descending aorta and with the foramen ovale flap moving.
- Right ventricle with the apex "filled in" by a piece of muscle called the moderator band.
- Two atria of similar size.
- Two ventricles of similar size, thickness and contractility (the right ventricle may be slightly larger).
- The interatrial and interventricular septum join the atrioventricular valves in the middle of the heart, suggesting the image of a cross, the "crux cordis."
- The interventricular septum should be intact and make an angle of approximately 45º with the midline of the body.
- Two atrioventricular valves with equal opening orifices. The insertion of the septal leaflet of the tricuspid valve is closer to the cardiac apex, resulting in a minimal difference in the level of implantation of the anterior leaflet of the mitral valve. Sometimes, this difference is quite subtle, resulting in great difficulties in excluding the diagnosis of atrioventricular septal defect and single AV valve junction.
- The interatrial septum may be seen with the foramen ovale and its flap, tilting with the LA.
- The pulmonary veins drainage in the left atrium should be identified in two-dimensional view and confirmed by colored Doppler or power Doppler.

Failure to obtain a normal four chamber view during the obstetric ultrasound scan is an absolute indication for fetal echocardiogram. Because the four-chamber view does not include the examination of the right and left ventricular outflows, important diseases such as transposition of the great arteries, tetralogy of Fallot (TOF), common truncus arteriosus, among others may be missed. Tables 2.2 and 2.3 show the different heart diseases commonly associated with normal and abnormal four chamber views, respectively.

**Table 2.2:** Heart diseases commonly associated with a normal four-chamber view

Tetralogy of Fallot
Transposition of the great arteries
Common truncus arteriosus
Anomalies of the aortic arch
Mild aortic and pulmonary valve stenosis
Perimembranous ventricular septal defect

**Table 2.3:** Heart diseases commonly associated with an abnormal four-chamber view

Mitral and/or aortic atresia
Tricuspid and/or pulmonary atresia
Ebsteins anomaly/tricuspid valve dysplasia
Atrioventricular septal defects
Large ventricular septal defects
Single ventricles
Severe aortic and pulmonary valve stenosis
Coarctation of the aorta
Total anomalous pulmonary venous return
Cardiomyopathies
Cardiac tumors

#### 2.2.3. Step 3 - 3^rd^ Level: Left Ventricular Outflow Tract

Starting from the four-chamber views, the left and right outflow tracts and respective arteries can be seen swiping the transducer toward the fetal head. The left ventricular outflow tract is the first identified in the middle of the heart and it directs toward the fetal right shoulder. In this view it is possible to observe the membranous continuity of the septum with the aorta, which rules out a possible overriding aorta or great artery commonly seen in tetralogy of Fallot, truncus arteriosus, and other complex anomalies.

#### 2.2.4. Step 4 - 4^th^ Level: Right Ventricular Outflow Tract

Swiping slightly the transducer up, the right ventricular outflow tract is reached. It is the most anterior structure of the heart and is exactly below the fetal sternum. It crosses aorta from right towards the left. The great arteries are symmetric at the beginning of gestation, but during the second and the third trimesters the pulmonary trunk is slightly larger than the aorta.

#### 2.2.5. Step 5 - 5^th^ Level: Three Vessels View

This is a special view that allows to analyze the spatial relationship of the pulmonary artery, the aorta and the superior vena cava (SVC). In this view the vessels are seen immediately after their ventricular origins. Important information should be obtained from the vessels: number - that should be three; position - SVC on the right, aorta on the middle and pulmonary artery on the left; size - SVC slightly smaller than aorta that should be slightly smaller than the pulmonary artery and finally, alignment - the SVC is more posterior, aorta is in the center and pulmonary artery is anterior.^19^ In this plane, the right and left bronchi are observed.

#### 2.2.6. Step 6 - 6^th^ Level: Three Vessel and Trachea View

Immediately above this plane, i.e., tilting the transducer a bit further in the cephalic direction, a view of two large arches connecting with the descending thoracic aorta is obtained. The one on the left is the ductus arteriosus that originates from the pulmonary artery and the other on the right is the aortic arch, both connecting with the descending aorta. This view makes a figure that suggests the letter V. The trachea appears as an anechoic structure surrounded by a hyperechoic line which corresponds to cartilage, being situated in front of the spine, slightly to the right.

**Figure 2.2:**
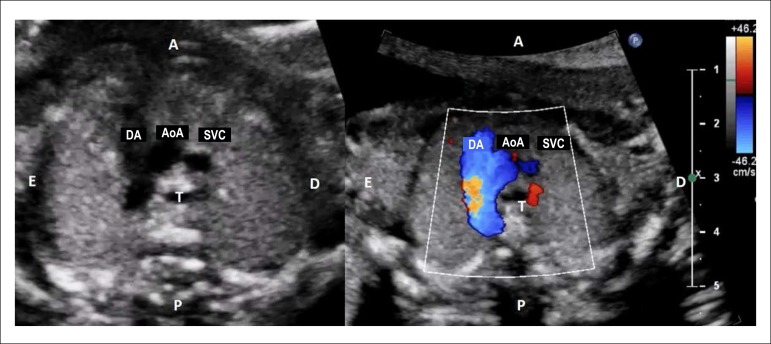
Aorta and pulmonary artery appear elongated, going toward the descending aorta. Both converge to the aorta forming an image similar to a V letter. The trachea is to the right of the aortic arch, demonstrating that the latter descends to the left. During color flow mapping, both arches are observed to have flow in the same direction, i.e., from the heart toward the descending thoracic aorta. AoA: aortic arch; DA: ductal arch; SVC: superior vena cava; T: trachea.

In this view, the aortic arch turns toward the left, which is defined exactly by its relation to the trachea. If the trachea is to the right of the aortic arch, the arch is turned toward the left and vice versa. It is worth highlighting that, the use of color flow mapping should be used during all screening steps and levels, and it is of particular importance during this final view. Both arches should present flow in the same direction, always directed from the heart toward the descending thoracic aorta (Figure 2.2).

### 2.3. Screening for Congenital Heart Disease During the First Trimester

Because CHD are the most common severe congenital defects and the least diagnosed by routine obstetric ultrasound, the challenge over recent years has been early screening methods for fetal heart disease, considering the fact that the majority of babies affected by heart disease are born to mothers who do not present the classic indications for fetal echocardiography.

Older studies have shown a sensibility of up to 40% in the detection of CHD in fetuses with increased nuchal translucency (NT), between weeks 11 and 14 of gestation (above the 99^th^ percentile). Focusing on fetuses with increased NT and normal karyotype, they demonstrated an incidence of heart disease 5 to 7 times greater in this group.^20-22^

The most recent literature shows a sensibility of about 13.5% for the detection of cardiac abnormalities, being NT ≥ 3.5 mm considered an indication for fetal echocardiography.^23-25^

Doppler flow analysis of the fetal cardiovascular system is also applied to screen CHD that may or not be associated with chromosome diseases. Several studies have argued that abnormal flow of the ductus venosus, i.e., the appearance of the reverse wave during atrial contraction ("a" wave) in fetuses with NT ≥ 3.5 mm increases the probability of CHD three-fold, whereas a normal flow pattern decreases the risk of heart disease by half.^21^ The presence of tricuspid regurgitation during the first trimester of pregnancy is highly associated with trisomy. When present in chromosomally normal fetuses, the risk of heart disease is observed to increase eight-fold. The etiology of tricuspid regurgitation in the first semester is uncertain; it is known only that it disappears concomitantly with the normalization of nuchal thickness.^24^

### 2.4. Fetal Echocardiography

Before beginning the examination, it is very important to obtain information regarding gestational age, previous obstetric history, possible maternal disease or use of medications that may increase the risk for CHD, and the formal indication for the study. This will provide the cardiologist with the possible risks for cardiac anomalies.

The ultrasound system may be specific for echocardiography or ultrasonography, provided with a preset for fetal heart/echocardiography. Convex (ultrasonography) or phased array (echocardiography) transducers allow to obtain good quality images, with the observation that the majority of convex transducers do not provide continuous Doppler, which may be useful in cases of valvular stenosis or regurgitation.

Volumetric transducers may allow better two-dimensional imaging in obese pregnant women and first trimester examination, but they are not essential in daily practice, being considered sophisticated technology not available in the majority of fetal scanning laboratories.

After 18 weeks gestation, all cardiac structures may be securely analyzed by the fetal echocardiogram except in cases of poor acoustic windows like obesity, polyhydramnios, oligohydramnios and others. The best images, however, are obtained between weeks 24 and 28, when the heart is larger in size, the fetus continues moving well, and the bones do not constitute a significant ultrasound barrier. It is worth highlighting that early evaluation of the heart may be performed either by transvaginal or transabdominal ultrasound (after week 14); this is usually indicated in pregnancies with high risks of fetal heart disease, especially when screening at the first trimester is indicative of cardiac anomaly.^24^

It is essential that the fetal cardiologist has a basic understanding of ultrasonography concepts, particularly regarding fetal status and position. Before beginning the evaluation of the heart, the position of the fetus must be determined, identifying right and left sides. The main marker of the fetal left side is the stomach. In the event of situs inversus or situs ambiguous, the stomach may be displaced, and should not be used as a marker of the fetal left side.

The best image of the heart is obtained from the abdomen, sliding the transducer slightly toward the thorax. Although it is also possible to obtain images from the front or the back of the baby, the images obtained from the back may be of inferior quality, especially during the last trimester, when the ossification of the ribs and the spine represents an important barrier to ultrasound passage. In this situation, to improve image quality, one may request patient to lie in left or right lateral decubitus position.

Polyhydramnios is a condition that may pose great difficulties to perform the examination, since the fetus may be too far from the transducer and move constantly. Perform measurements and place the Doppler sample volume in place to obtain the usual traces may be really challenging. In situation like this, the fetus may be brought closer to the transducer, if the patient lies or her knees and elbows. Maternal obesity also poses difficulties to the technical quality of the study and it is often needed a low-frequency transducer, sometimes such as those used for adult echocardiography with more vigorous compression to the maternal abdomen.

Once the fetal heart has been identified, only small movements of the transducer are necessary to analyze all the cardiac structures. Considering that the fetal heart is relatively far from the transducer, small movements mean big changes in angle. Fetal echocardiography is considered complete when the heart has been examined from all possible views and planes, including the projections obtained in a conventional postnatal echocardiogram.

**Figure 2.3:**
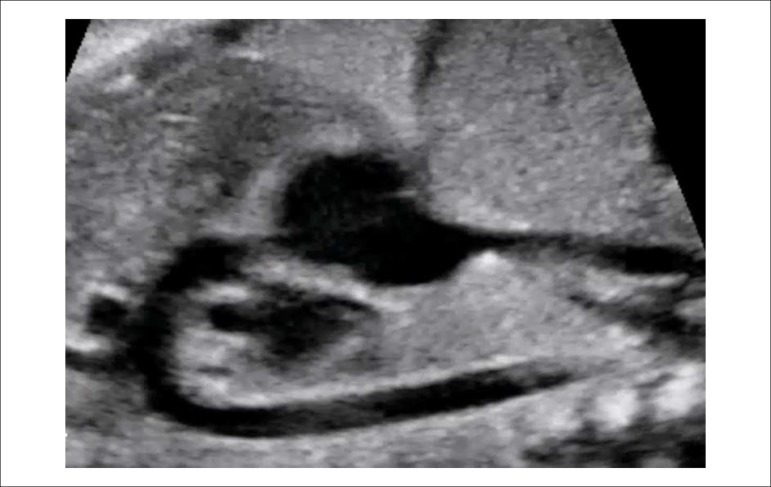
Long axis view of the aortic arch. The shape of the aortic arch is similar to a cane.

**Figure 2.4:**
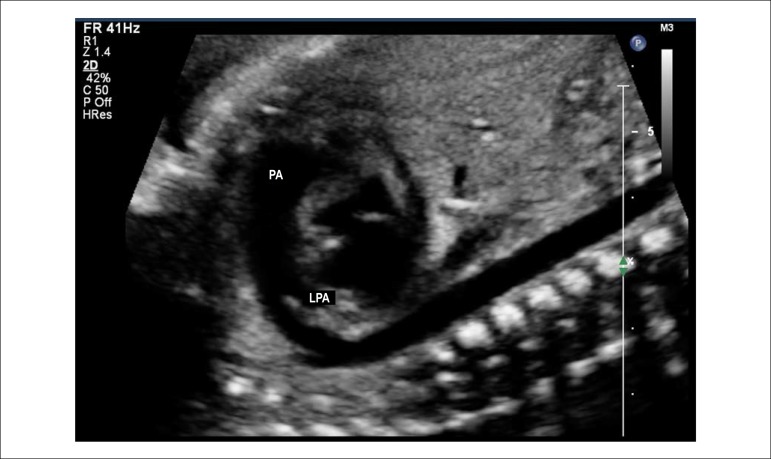
Longitudinal plane slightly anterior and to the left of the fetus, showing the long axis view of the ductal arch. The ductal arch has a different angle than the aortic and looks like a golf club. LPA: left pulmonary artery; PA: pulmonary artery.

**Figure 2.5:**
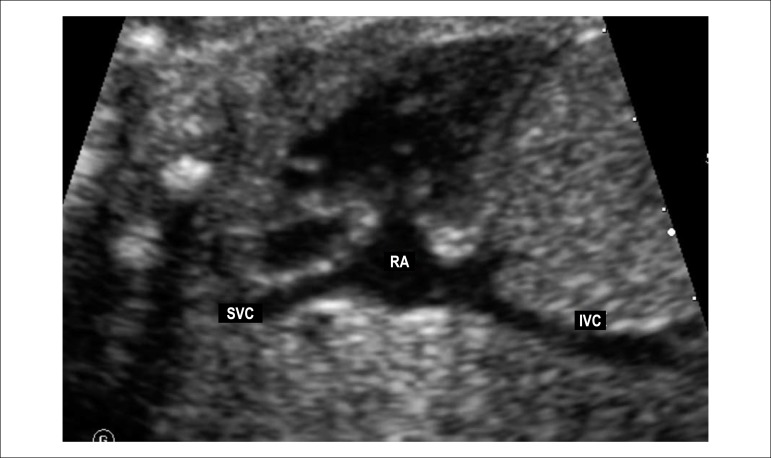
Longitudinal fetal plane tilting posteriorly, showing the bicaval view. IVC: inferior vena cava; RA: right atrium; SVC: superior vena cava.

**Figure 2.6:**
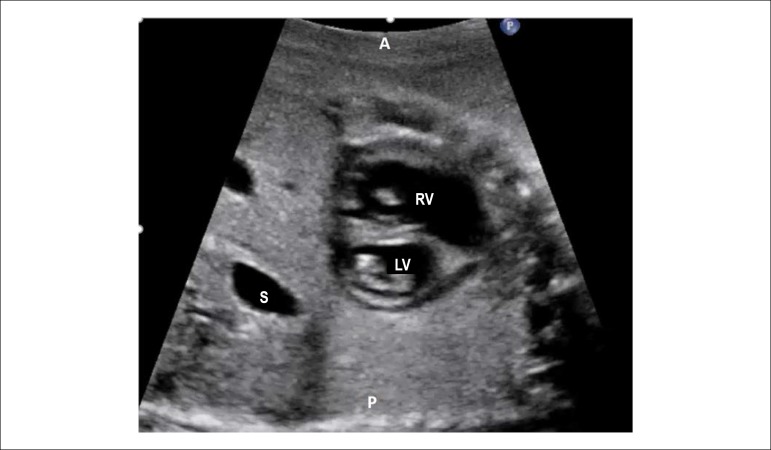
Short-axis of the ventricles. In this plane it is possible to analyze the position of the papillary muscles of the right and left ventricles. It is also of great utility in detecting subtler forms of atrioventricular septal defect when it is presented with two valvular orifices. A: anterior; P: posterior; LV: left ventricle; RV: right ventricle; S: stomach.

**Figure 2.7:**
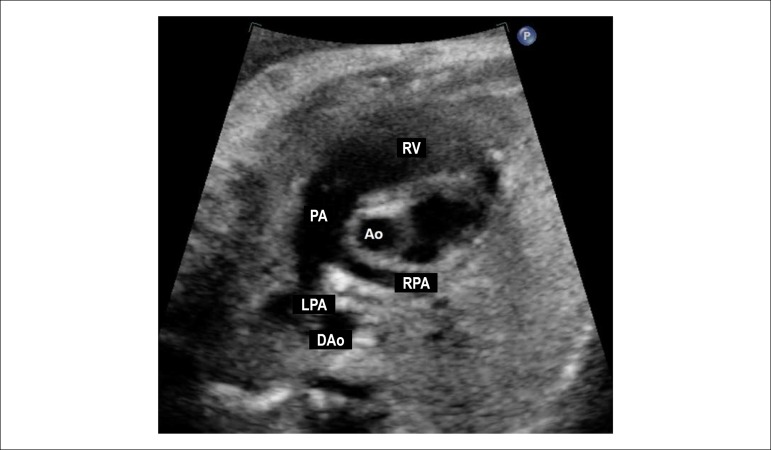
Short axis view of the great vessels. This plane shows the relationship between the great arteries, with the aorta being in the center of the heart and posteriorly and the right ventricular outflow tract surrounding the aorta anteriorly. This is an excellent plane for identifying perimembranous ventricular septal defects and pulmonary obstructions due to the anterior deviation of the infundibular septum observed in the tetralogy of Fallot. Ao: aorta; DAo: descending aorta; LPA: left pulmonary artery; PA: pulmonary artery; RPA: right pulmonary artery; RV: right ventricle.

Differently from the recommendations for obstetric screening for cardiac malformations, fetal echocardiography must include transverse and longitudinal views of the fetus, what guarantees different sights of the same structure.^18^ The following images should also be included to the 6 transverse levels: long axis of the aortic and ductal arches (Figures 2.3 and 2.4), bicaval view (Figure 2.5), and short axis of ventricles and great vessels (Figures 2.6 and 2.7).

### 2.5. Imaging Techniques Used on Fetal Echocardiography

Experienced imaging professionals, such as ultrasound specialists, radiologists, or echocardiographers may evaluate the fetal heart with high diagnostic accuracy. However, knowledge of the anatomical, physiological and possible therapeutic algorithms are essential to obtain the most accurate information and counsel the family. To avoid missing information, the international medical societies of echocardiography and ultrasound have established the obligatory contents of a complete fetal echocardiogram.

Based on the AHA guidelines published in 2014, mandatory elements (Class of Recommendation I), elements whose inclusion is reasonable (Class of Recommendation IIa) or may be reasonable (Class of Recommendation IIb) were distinguished (Table 2.4).^17^

**Table 2.4:** Fetal echocardiogram mandatory, optional, and recommended elements

Essential, mandatory elements (Class I)	
Two-dimensional echocardiographic anatomy	Cardiovisceral situs
	Cardiac position
	Pericardial effusion
	Systemic and pulmonary venous connections
	Atrial morphology
	Atrial septal morphology
	Atrioventricular connection
	Ventricular morphology, size, and comparative analysis of the ventricular sizes
	Ventricular-arterial connection
	Atrioventricular valves morphology, size, and comparative analysis of the valvular sizes
	Semilunar valves morphology, size, comparative analysis of the valvular sizes
	Ventricular septal morphology
	Great arteries anatomy, size, and comparative analysis of the great arteries sizes
	Three vessels and three vessels and trachea views
	Aortic arch morphology
	Ductal arch morphology
	Proximal pulmonary arteries
Color doppler	Superior and inferior vena cavae
	Foramen ovale
	Atrioventricular valves/ventricular inflows
	Interventricular septum
	Semilunar valves/ventricular outflows
	Ductus venosus
	Pulmonary veins
	Great arteries
	Left and right pulmonary arteries
	Aortic and ductal arches
Pulsed-wave doppler	Atrioventricular valves/ventricular inflows
	Semilunar valves/ventricular outflows
	Ductus venosus
	Umbilical vein
	Umbilical artery
	Pulmonary veins
	Great arteries
	Ductal arch
Heart rate and rhythm assessment	
**Optional elements (classes IIa and IIb)**	
Cardiac and general biometry	Cardiothoracic ratio
	Atrial dimensions
	Ventricular dimension
	Atrioventricular valve diameters
	Semilunar valve diameters
	Ascending aortic and main pulmonary artery diameters
	Aortic and ductal arch diameters
	Branch pulmonary artery diameters
	Fetal biometry
Color doppler	Umbilical vein and arteries
Pulsed-wave doppler	Superior and inferior vena cavae
	Right and left pulmonary arteries
	Middle cerebral artery
Other doppler modalities	Continuous-wave doppler
	Tissue doppler
Additional cardiac function indexes	Ventricular shortening fraction
	Myocardial performance index
	Calculation of cardiac output

## 3. Stratification of Centers that Work with Fetal Cardiology and their Potential Therapeutic Facilities

Congenital heart diseases are the most frequently malformations related to morbimortality in infancy, especially during the prenatal period.^26^ Its incidence has been estimated as 6 to12 cases per 1,000 live births. During fetal life, it may be up to 5 times higher, being the difference justified by fetal losses.^27-29^ Approximately 50% of cases have early hemodynamic consequences, requiring catheter or surgical interventions during the first year of life.^17^ Extracardiac malformations may be observed in up to 50%, further increasing pre- and postoperative morbimortality.^30^ It is worth highlighting that, in developed countries, treatment for CHD compared to other congenital anomalies, have the highest hospital costs.^31^

Over the past years, first-trimester ultrasounds and, widespread use of fetal echocardiography have contributed to increase the rates of fetal diagnosis of CHD and consequently, improve perinatal outcome.

However, fetal echocardiography has not become universally available in Brazil yet, with the majority of professionals trained in Fetal Cardiology being concentrated in the South and Southeast Regions and a more restricted number in the North, Northeast, and Central-West Regions. In the states located in these latter regions, the availability of this tool is mainly restricted to the capital cities and is of low availability in Brazil's public healthcare system (*Sistema Único de Saúde*, SUS).^32-34^

There is a decreasing tendency of Brazil's infant mortality rates over the last years, with a 77% decline over 22 years, from 62 deaths per 1,000 live births in 1990 to 14 per 1,000 in 2012.^35^ Deaths during the first year of life represent 90% of mortality in the 0-4 age group, with 68% occurring between 0 and 28 days. Congenital cardiac anomalies have been identified as responsible for a significant part of these rates, especially during the neonatal period.^35^ It is estimated that there are approximately 25,700 new cases of CHD per year in Brazil, which are distributed regionally as follows: 2,758 cases in the North Region; 7,570 in the Northeast; 10,112 in the Southeast; 3,329 in the South, and 1,987 in the Central West.^36^ In 2010, the Live Births Information System (*Sistema de Informação sobre Nascidos Vivos*, SINASC) of the Ministry of Health, had 1,377 cases of live births with CHD notified. This represents only 5.3% of the estimated number.^36^

There are currently approximately 40 services accredited by the Ministry of Health to perform pediatric cardiac surgery, with a rather heterogeneous distribution, concentrated mainly in the South and Southeast Regions (62%). In accordance with 2002 data from the Brazilian Unified Health System's Department of Informatics (*Departamento de Informática do Sistema Único de Saúde*, DATASUS), the deficit in cardiac surgery for CHD in the North and Northeast Regions was 93.5% and 77.4%, respectively.^32,33^ As the implantation of Fetal Cardiology is directly related to pediatric cardiac surgery services, the current situation in Brazil, with respect to fetal diagnosis, continues to be considerably heterogeneous.^34^

According to their potential therapeutic facilities, Fetal Cardiology centers were stratified on three specific levels:

- **Level 1:** Centers that can diagnose structural and functional fetal cardiac anomalies, make the follow-up of the affected fetuses and, stablishe the delivery planning according to the fetal heart disease.
- **Level 2:** Centers where, in addition to the fetal diagnoses of structural and functional fetal cardiac anomalies, have a multidisciplinary team with obstetricians, pediatric cardiologists, interventional cardiologists, and pediatric cardiac surgeons, and can provide the postnatal therapy.
- **Level 3:** Centers where, in addition to diagnosis and follow-up of the affected fetuses, have a multidisciplinary team with obstetricians, pediatric cardiologists, interventional cardiologists, and pediatric cardiac surgeons and provide invasive intrauterine interventions.

Currently, in Brazil, the majority of Pediatric Cardiology centers are considered levels 1 or 2. Intrauterine interventions are restricted to a very small number of fetuses with very specific pathologies and particularities that benefit from fetal therapy. For this reason, the existence of more than 1 or 2 centers with these characteristics in Brazil is not justified.

It is clear that Brazil needs more Pediatric and Fetal Cardiology centers as well as increase the number of cardiac surgeries and percutaneous interventions. Nevertheless, due to various political and environmental issues, these changes will only occur in medium to long term.

Aiming to maximize referrals of fetuses with CHD to the existing centers, it is mandatory that all professionals involved in the screening of CHD know how to refer the patient to the appropriate care centers.^37,38^ When fetuses with CHD are identified in places where there is no appropriate care, the doctor should promptly look for help to refer the patient to a specialized center according to the regulatory flow of the state. If the state has no specific hospital do refer the patient, the local health system should ask for outside treatment (tratamento for a de domicílio -TFD) which will look for the closest specialized center to take care of the mother and the fetus. This process is nowadays regulated by CNRAC (central nacional de regulação da alta complexidade) since directive instructions of the Ministry of Health to organize the health care for high-risk pregnancies were published. It is emphasized here that the high-risk pregnancies are "those in which the life or health of the mother, the fetus, or the newborn has higher chances of being affected when compared to the general population."^39^

When fetal cardiologist is dealing with a case of fetal heart disease, he or she needs to define whether there is any need of prenatal intervention or whether the treatment has to be started immediately after birth and if the patient needs to be referred to center levels 2 or 3 available in our country, reminding that not all the centers considered level 2 can treat all types of neonatal anomalies. It is known that HLHS and its variations, for example, have an extremely high fetal incidence, whereas few centers in our country have satisfactory operative results for this anomaly.

**Figure 3.1:**
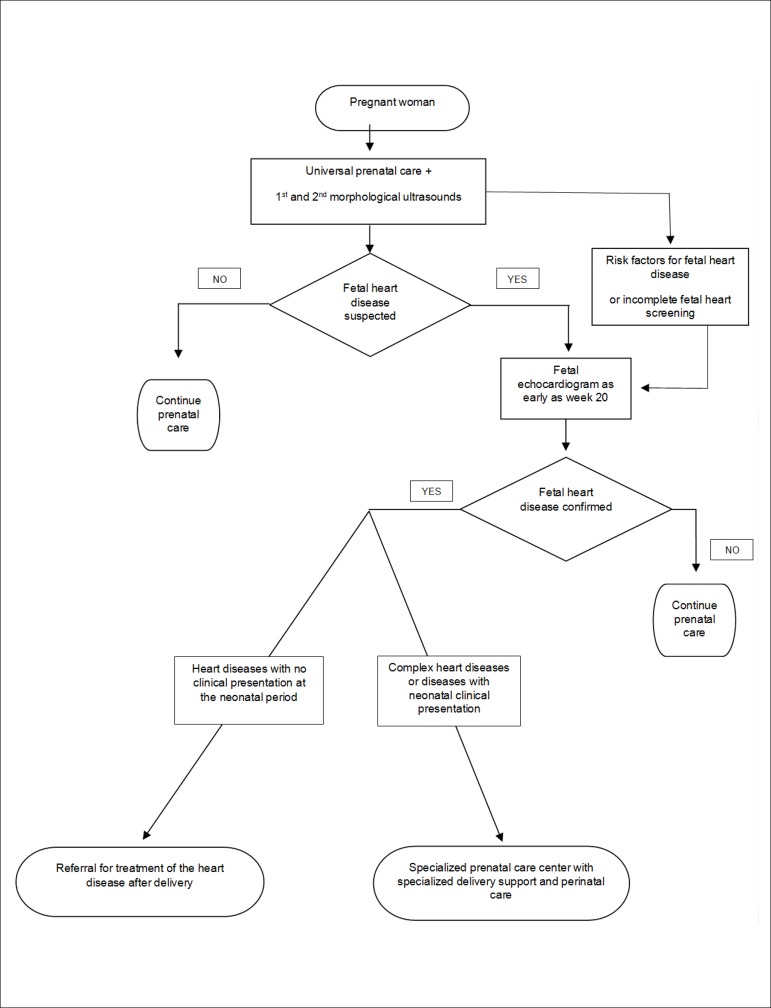
Fetal care flowchart according to the specific fetal heart condition. USG: ultrasound.

The Figure 3.1 is a flowchart that standardizes the specific care according to the fetal heart disease.

## 4. Classification of Fetal Heart Disease

With the development of fetal medicine as a medical subspecialty and with the recent advances in the ultrasound imaging, the detection of fetuses with congenital malformations has become increasingly frequent, making earlier treatment possible with significant reduction of fetal and neonatal mortality.^40^ With prenatal diagnosis, diseases with potential risk to have hemodynamic compromise in utero and/or in the neonatal period can be followed up and have the specific pre and postnatal care planned. Taking into account the characteristics of the fetal circulation, it is particularly important to recognize the behavior of the different heart diseases in utero and after birth, identifying those that will require any kind of treatment (use of medications or invasive procedures) or anticipation of the childbirth.^41^

Fetal cardiac disease may be classified as structural or functional. The majority of the structural heart diseases do not have hemodynamic compromise in utero due to the fetal circulation physiology. Clinical manifestations will occur after birth, when the physiological intracardiac shunts close. Cardiomyopathies, conditions like high output fistulas, significant abnormalities of the cardiac rhythm and restricted foramen ovale, ductal constriction or absent ductus venosus may also occur in utero and compromise the fetal hemodynamic requiring prenatal treatment.

It is important to highlight the importance of a multidisciplinary team involved in the care of fetuses affected by heart diseases, since genetic syndromes or severe extracardiac malformations may be associated and significantly increase postnatal mortality.

For these reasons, fetal heart diseases were classified into 3 groups according to possible clinical presentation and in utero hemodynamic manifestations and were separated in groups A - structural and B - functional (Table 4.1).

**Table 4.1:** Classification of fetal anomalies according to fetal outcome

Group	Fetal outcome
I	Heart diseases without fetal hemodynamic compromise A. Structural B. Functional
II	Heart diseases with fetal hemodynamic compromise A. Structural B. Functional
III	Heart diseases with poor postnatal prognosis

### 4.1. Group I − Heart Diseases without Fetal Hemodynamic Compromise

#### 4.1.1. Structural

This group includes simple or complex cardiac defects that do not usually present progression or hemodynamic decompensation during the fetal period and, thus, do not require treatment during pregnancy and do not change obstetric management. The main example of this group are diseases with left-to-right shunt, including atrial, ventricular and atrioventricular septal defects, and aortic to pulmonary window; heart diseases with mild obstruction of right or left outflow tracts, such as pulmonary valve stenosis, aortic stenosis, and localized coarctation of the aorta; and complex CHD such as TOF with mild pulmonary flow obstruction, corrected transposition of great arteries, double outlet right ventricle, and univentricular hearts without obstructions or with mild obstructions to systemic and pulmonary outflow tract flows.

#### 4.1.2. Functional

This group includes cardiac rhythm abnormalities such as isolated supraventricular extra beats and mild isolated tricuspid regurgitation.

### 4.2. Group II − Heart Diseases with Fetal Hemodynamic Compromises

#### 4.2.1. Structural

This group includes cardiac defects that may compromise the development of cardiac structures throughout gestation, such as critical or total obstruction of the ventricular outflows,^42,43^ defects that potentially trigger heart failure due to the presence of severe valvular insufficiency,^44^ and anomalies that require patent foramen ovale to divert flow from one chamber to the other (atrioventricular valves atresia or stenosis), being the main examples HLHS and tricuspid atresia.^45^ This group requires special attention, and some of the cases may benefit from a fetal cardiac intervention to increase the blood mixture at the atrial level (see the Fetal Interventions Chapter).^44^

#### 4.2.2. Functional

Primary fetal myocardial dysfunctions have various etiologies. They may be caused by myocarditis (usually viral), structural changes in myocardial fibers (noncompacted myocardium, deposit diseases such as mucopolysaccharidoses or glycogenoses), and they may be related to maternal diabetes and genetic conditions.^46,47^ Severe cardiac arrhythmias, such as sustained tachyarrhythmias and complete atrioventricular block (CAVB) lead to cardiac chambers dilation, atrioventricular valves regurgitation, and myocardial dysfunction.^48^ Tachyarrhythmias are considered emergencies in Fetal Cardiology due to the risk of hydrops and fetal death; being the majority of cases possible to be treated with antiarrhythmic medication.^49^

Cardiac tumors are rare. The most prevalent in fetal life is rhabdomyoma. They may be single or multiple and their dimensions increase during fetal life.^50^ Serial echocardiograms are indicated because of the risk of arrhythmias, ventricular outflow obstructions, or cardiac structures compression. Functional abnormalities of the intracardiac shunts may imbalance the distribution of fetal blood flow. Ductal arteriosus constriction, the most frequent, will be detailed discussed in a subsequent chapter.^51^ Restrictions of blood flow through the foramen ovale and agenesis of the ductus venosus are rare conditions. Both evolve with right chamber dilation and may lead to fetal heart failure.^52,53^ High-output fistulas may lead to cardiac chambers dilation and dysfunction and fetal hydrops. The most frequent are Galen's vein aneurysm, hemangioma, hepatoblastoma, pulmonary arteriovenous malformation, vascularized tumors such as sacrococcygeal or cervical teratoma, and the twin-twin transfusion syndrome.^54,55^ Severe anemia resulting from viral infection or blood type incompatibilities may lead to heart failure. Fetal hemodynamics may also be compromised by extrinsic fetal heart compressions, such as diaphragmatic hernia, pulmonary cystic adenomatoid malformation, and pericardial tumors. This group needs serial fetal echocardiograms, ideally biweekly, and this interval may be reduced if needed. The cardiovascular profile score, published by Huhta et al should be employed to establish the outcome.^56^

### 4.3. Group III − Fetal Heart Diseases with Limited Postnatal Prognosis

This group corresponds to very severe heart diseases in which, any therapeutic measurements will result in nearly 100% chance of death. It includes severe forms of left atrial isomerism associated with CAVB, obstruction of both ventricular outflows tracts and myocardial disease, critical obstructive malformations associated with noncompacted myocardium, the worst spectra of Ebstein's anomaly or tricuspid valve dysplasia associated with lung hypoplasia, left ventricular aneurysms with fetal congestive heart failure, and heart diseases associated with chromosomal disorders with limited prognosis (trisomies of 13 and 18). In this group, multidisciplinary follow-up, including psychological support for parents must be prioritized, but delivery may be in a hospital with basic support (Table 4.2).

**Table 4.2:** Distribution of fetal heart diseases according to their classification

Group	Cardiac anomalies
IA	Left to right shunt heart diseases: ASD, VSD, AVSD, and Ao-P window Diseases with mild outflow tract obstructions: PS, AS, and CoA Complex congenital heart diseases without significant obstructions of systemic or pulmonary outflow tracts: TOF, complex TGA, DORV, univentricular hearts, and CTGA
IB	Isolated extrasystoles; mild, isolated TR
IIA	Heart diseases with critical obstruction of systemic or pulmonary outflow tracts: PAIVS, Critical PS, Critical AS, and HLHS Heart diseases that need interatrial shunt: HLHS and variations, TGA, and TA Heart diseases with severe valve insufficiencies: Ebsteins anomaly and tricuspid valve dysplasia, pulmonary valve agenesis, severe primary or secondary MR, secondary TR, and truncal valve insufficiency
IIB	Cardiomyopathies and myocarditis, arrhythmias, obstructive tumors, extrinsic compressions, (CDH and CCAM), ductal constriction, restrictive foramen ovale, ductus venosous agenesis, AVMs, TTTS, and twin gestation with 1 acardiac fetus
III	Severe chromosomal disorders; multiple malformations, cardiac defects that are not correctable, very severe forms of Ebsteins anomaly or tricuspid valve dysplasia with lungs hypoplasia, LV aneurysms, or diverticula associated with fetal hydrops

Ao-P: aortic to pulmonary; AS: aortic stenosis; ASD: atrial septal defect; AVMs: arteriovenous malformations; AVSD: atrioventricular septal defect; CCAM: congenital cystic adenomatoid malformation; CDH: congenital diaphragmatic hernia; CoA: coarctation of the aorta; CTGA: corrected transposition of great arteries; DORV: double outlet right ventricle; HLHS: hypoplastic left heart syndrome; LV: left ventricle; MR: mitral regurgitation; PAIVS: pulmonary atresia and intact ventricular septum; PS: pulmonary stenosis; TA: tricuspid atresia; TGA: transposition of great arteries; TOF: tetralogy of Fallot; TR: tricuspid regurgitation; TTTS: twin-twin transfusion syndrome; VSD: ventricular septal defect.

## 5. Management of the Main Fetal Heart Diseases

One of the main challenges for the ultrasound specialist and pediatric cardiologist is to know exactly what to do when they face a fetus with CHD. Because of the fetal physiology characteristics, the majority of cardiac anomalies have a benign outcome in utero. However, at birth, they may become devastating, and require specific treatment immediately after the umbilical cord clamping. On the other hand, mild fetal cardiac abnormalities may be overvalued and lead unnecessary attitudes regarding gestation and delivery conduction, just because of the lack of knowledge of the real impact of the anomalies to the baby's health after birth. Although prenatal diagnosis has been possible for more than 40 years, understanding the behavior of CHD during the pre- and postnatal periods has become clearer over the past last years, thanks to the diagnostic accuracy improvements and to the introduction of fetal therapy that lead to progressive understanding of their natural and modified history. For these reasons, in order to guide the need of delivery and/or treatment in specialized centers, cardiac anomalies were separated into several groups according to their perinatal outcome: with and without hemodynamic compromise, with and without in utero progression, and possible postnatal outcome (Tables 5.1 to 5.8).

**Table 5.1:** Group IA. Structural fetal heart diseases without in utero hemodynamic compromise, which do not require immediate neonatal care. Class of recommendation/level of evidence: IB.^17^,^41^,^57^-^59^

Heart disease	In utero outcome	In utero follow up	Delivery	Postnatal assessment
VSD AVSD ASD Ao-P window	Stable	Repeat the study a few weeks before birth is recommended	Delivery type according to obstetric indication Level 1 center	Maternity ward or outpatient clinic

Ao-P: aortopulmonary; ASD: atrial septal defect; AVSD: atrioventricular septal defect; VSD: ventricular septal defect.

**Table 5.8:** Group III. Fetal heart diseases associated with genetic syndromes or extracardiac malformations. Class of recommendation/level of evidence: IIb C.17,41,57-59

Heart disease	In utero outcome	In utero follow up	Delivery	Postnatal assessment
Multiple malformations Associative syndromes Trisomies Triploidy Other genetic anomalies	May evolve with fetal hydrops depending on the genetic of extracardiac anomaly	Depends on fetal or neonatal viability and extracardiac anomalies prognosis	For non-viable fetuses or newborns, delivery may be in a level 1 center, preferably by spontaneous vaginal birth. For viable fetuses or newborns, delivery may be vaginal or programmed C-section in a level 2 or 3 center Consider palliative care team support	Cardiac management according to prognosis of associated anomalies or chromosome diseases

## 6. Fetal Ductal Constriction: Treatment and Prevention

Fetal circulation has specific characteristics, differing morphologically and functionally from extrauterine circulation. Anatomically, the ductus arteriosus is part of the right ventricular outflow tract, playing a essential role in directing blood flow to lower portions of the fetus. Basically, the ductus arteriosus carries 80-85% of right ventricular output to the descending aorta.^60^ It's histological structure is composed of a thick muscle layer, which increases with gestational age. Its constrictive mechanism is facilitated by the circumferential orientation of muscular fibers, especially those of the external layer.^61^ Due to these histological characteristics, its patency is measured by multiple factors. Luminal abnormalities may cause severe fetal and neonatal complications, such as heart failure, hydrops, persistent neonatal pulmonary hypertension, and death.^62-64^

Typically, maternal use of indomethacin and/or other anti-inflammatory medications interferes with the metabolism of prostaglandins (PG), causing ductal constriction.^65-67^ Many causes of ductal constriction and neonatal pulmonary hypertension, however, are not related to the use of these substances and are classified as idiopathic.^68^

A growing amount of evidence has recently shown that herbs, fruits, nuts, and a wide variety of substances commonly consumed as part of a daily diet affect the inflammatory cascade, culminating in reduced PG synthesis.^69,70^ This anti-inflammatory action, especially of polyphenols, when ingested during the third trimester of gestation, influences the dynamics of the fetal ductus arteriosus.^71-78^

### 6.1. Prevalence, Diagnosis, Clinical Consequences, and Prognosis of Fetal Ductus Arteriosus Constriction

The prevalence of ductal constriction detected in a convenience sample of 16,079 records of fetal echocardiograms performed during the third trimester of gestation, over a period of 11 years, excluding all other concomitant anomalies, in Porto Alegre, Rio Grande do Sul, Brazil was 2.7% (435 cases). During this period, there were 207,323 live births; the sample thus represented 7.75% of births.^79^

Experimental studies have shown that fetal ductal constriction results in an increase in the medial layer of the pulmonary artery, which leads to a secondary increase in pulmonary vascular resistance in utero.^80^ Thus, the majority of studies on persistent pulmonary hypertension are based on the experimental model of fetal ductal constriction induced by the administration of indomethacin.^81^ Moderate or chronic ductal constrictions lead to pulmonary hypertension due to the increase in the medial layer and consequent increase in pulmonary artery constriction. This sustained increase in right ventricular afterload may lead to morphological, functional, and histological modifications in the right ventricular myocardium.^82^ Ventricular dysfunction in cases related to maternal medication ingestion may be completely reverted following its interruption. The persistence of the dysfunction, however, may even lead to myocardial ischemia with papillary dysfunction.^80,83,84^ Fetal cardiac dysfunction is described as one of the characteristics of fetal ductal closure and, in severe cases, the possibility of anticipation the childbirth should be considered, once fetal pulmonary maturity is reached.^85^ Postnatal clinical outcome depends on the severity of in utero right ventricular failure and response to the increased pulmonary vascular resistance.^86^

Long-term prognosis is uncertain; however, in cases with favorable initial outcome, there usually are no chronic complications. Nevertheless, after fetal heart failure, functional modifications may persist during the neonatal period, even in those with benign outcome.

Echocardiographic diagnosis of fetal ductal constriction is based on the presence of turbulent flow in the ductus, with an increase in systolic velocity (> 1.4 m/s), increase in diastolic velocity (> 0.3 m/s), and decrease in pulsatility index (PI) (< 2.2). In the first publication, the cutoff point for PI was 1.9.^87^ Recent studies, however, have considered a higher threshold.^78,88^ With the increased afterload secondary to ductal constriction, the heart shows symptoms of growth in earlier stages, hypertrophic response, with hyperplasia (substituted by apoptosis), increased right chamber proportions, increased pulmonary artery to aorta ratio, and interventricular septum bulging into the left ventricle.^89,90^ It is important to highlight that the diagnosis of ductal constriction and the evaluation of its severity cannot be established solely in terms of categorical variables of the "yes/no" sort, but are based rather on continuous variables, with a spectrum of circulation compromise (mild, moderate, or severe) which has been summarized in Table 6.1.

**Table 6.1:** Diagnostic criteria and classification according to the severity of ductal constriction

Criteria	1 point each	2 points each	3 points each
Systolic velocity, m/s	1.401.69	1.701.99	≥ 2.00
Diastolic velocity, m/s	0.300.34	0.350.39	≥ 0.40
Pulsatility index	2.22.1	2.01.9	≤ 1.8
RV:LV ratio	1.301.59	1.601.79	≥ 1.80
PA:Ao ratio	1.301.59	1.601.79	≥ 1.80
Septal bulging to the left	0 +/4	++/4	+++/4 ++++/4
Tricuspid regurgitation	0 +/4	++/4	+++/4 ++++/4

Ao: aorta; LV: left ventricle; PA: pulmonary artery; RV: right ventricle.

The scores are classified as followed:

Mild constriction: 3-7 points, the first 3 criteria being required

Moderate constriction: 8-14 points, the first 3 criteria being required

Severe constriction: > 15 points, the first 3 criteria being required.

As the vasoconstrictor effect in the ductus arteriosus is dose-dependent,^91^ the disappearance of hemodynamic abnormalities and non-development of fetal/neonatal cardiac dysfunction are common after the use interruption of constrictor substances.^89,92-95^ Even in severe cases of ductal constriction following use of substances that inhibit PG, their use interruption reduces systolic and diastolic ductal velocities, with improvements of the abnormal hemodynamics.^89^ There are no reports of important spontaneous reversal of ductal constriction without the removal of the causal factor.

In more severe cases, preterm delivery may be necessary, with immediate neonatal cardiopulmonary resuscitation measures. Although the relationship between the duration of the prenatal condition of ductal constriction and the prevalence and severity of neonatal pulmonary hypertension has yet to be defined, ideally it should be as short as possible. The moment of preterm delivery, thus, takes into account fetal pulmonary maturity, the severity of the of ductal constriction presentation and its progressive nature.^62^ To allow for recovery and early resolution of the process, it is obviously crucial to remove the cause immediately.

### 6.2. The Role of Anti-Inflammatory Substances in the Genesis of Fetal Ductal Constriction

The action of non-steroidal anti-inflammatory drugs (NSAID) results from PG synthesis inhibition caused by the inactivation of the cyclooxygenase 1 (COX-1) and 2 (COX-2) enzymes.^96^ This inhibitory effect reduces the formation of PGG2 and PGF2.^97,98^ The use of this class of medication for treating premature birth, preeclampsia, and restricted growth in utero has made it possible to evaluate its effects on COX and ductal constriction.

Indomethacin is the most studied NSAID medication. Its effect on COX is reversible after excretion.^99,100^ It crosses the placental barrier freely, as early as in the second gestational trimester.^101^ Fetal response to indomethacin, however, is individual, varying in studies with twin fetuses.^102^ Reports of constrictions before 27 weeks gestation are rare; however, they have occurred as early as week 22^nd^.^83^ Other PG synthesis inhibitors are involved in fetal ductal constriction, with well documented dose-dependent effects, for example, in dipyrone, paracetamol, scopolamine, fluoxetine, paroxetine and sertraline.^66,91,103-111^

Glucocorticoids also affect ductal patency. Their effects occur through the reduction of PG formation and ductal sensitivity to PGE2, with dose-dependent effects.^112,113^ Concomitant use with indomethacin has a synergetic effect that duplicates the incidence of fetal ductal constriction.^114^

### 6.3. Anti-Inflammatory and Antioxidant Action of Polyphenols

The main action of phenolic compounds or polyphenols is described in the literature as anti-inflammatory and antioxidant, demonstrating positive effects on cardiovascular health, cancer, diabetes, and neurodegenerative diseases.^115-117^

The antioxidant capacity of these compounds is essential to the organism in neutralizing the action of oxygen-reactive species,^118^ which, when produced excessively and not destroyed by endogenous defense, may interact with DNA, proteins, and lipids, culminating in the development of diseases such as cancer.^119,120^

Polyphenols play an important role in inhibiting the inflammatory cascade, with actions similar to that of NSAID, and are able to interfere with PG synthesis. The inflammatory cascade is initiated by the activation of phospholipase A2 (PLA2), stimulated, for example, by compounds such as thrombin, bradykinin, or epinephrine, upon membrane receptor binding. Activated PLA2 hydrolizes arachidonic acid (AA), or other similar polyunsaturated fatty acids, from membrane phospholipids. AA, in its turn, through the action of the COX-2 enzyme, initiates the formation cascade of PG and thromboxane (TX). Some NSAID, such as indomethacin, for example, inhibit the inflammatory cascade via inhibitory action of COX-2, a mechanism that has been studied in order to explain the similar effect of polyphenols in this process.

Polyphenols have their anti-inflammatory effects through a variety of molecular targets, which may be divided into 2 pathways: AA-dependent and AA-independent. COX, lipoxigenase, and PLA2 are AA-dependent inflammatory mediators. The activation of these proteins leads to the release of AA (a starting point for general inflammatory response) which promotes the release of pro-inflammatory molecules.^114^ On the other hand, nitric oxide synthase (NOS) nuclear factor-kappa B (NF-kB), and peroxisome proliferator activated receptor (PPAR) promote inflammation through AA-independent pathways.

### 6.4. Summary of Evidence for Ductal Constriction Management

A cornerstone of treating and preventing ductal constriction during fetal life is the reduction of fetal exposure to agents that interfere with the biosynthesis of PGE1, and PGE2.

The metabolic chain of PG production can be inhibited on different levels, such as in the decrease of AA production from phospholipids, by PLA2 inhibition, as is the case with corticosteroids, in the reduction of the transformation of AA to PGG2, measured by inhibition of COX-1 and COX-2, by maternal use of NSAID or consumption of polyphenol-rich foods, and by the inhibition of isomerase, which is responsible for the synthesis of PG, TX, and prostacyclin.

The inhibitory effect of NSAID on PG biosynthesis has been broadly demonstrated. Meta-analysis conducted in a systematic review of 25 randomized clinical trials, which evaluated the risk of fetal ductal constriction in pregnant women exposed and not exposed to NSAID, concluded that the risk of ductal constriction is 15 times greater in acutely exposed fetuses.^66^

Multiple randomized clinical trials, systematic reviews, and meta-analyses have established that polyphenols, in the various forms in which they are present in food, have a definite anti-inflammatory and antioxidant action, which culminates in the inhibition of circulating PG, with diversified clinical outcomes.

In 2015, the International Federation of Gynecology and Obstetrics (FIGO) published its official recommendations for gestational nutrition. One point in the section, "Exposures to avoid" reads:

"In late pregnancy, women **should avoid** high intakes of herbal teas and polyphenol-rich foods, which have been associated with effects on the fetal ductus arteriosus brought about by inhibition of prostaglandin synthesis." (italics ours)^106^

Specifically regarding the results of "abnormal ductal flow and ductal constriction" in fetuses exposed to a maternal diet rich in polyphenols, studies developed in Brazil, on all levels of the evidence pyramid, from experimental to case control studies, have unequivocally demonstrated the following:

- Consumption of green tea, yerba mate, and grape juice, which are sources of high concentrations of polyphenols, causes ductal constriction in experimental models of sheep fetuses in the final trimester of gestation.^121^
- There is a cause-effect relationship between maternal consumption of green tea and ductal constriction during the third trimester of gestation in experimental models of sheep fetuses.^74^
- High maternal consumption of polyphenols induces fetal ductal constriction in sheep, with increased urinary excretion of total polyphenols and abnormalities in oxidative stress biomarkers, which characterize the anti-inflammatory and antioxidant actions of polyphenols.^122^
- An experimental single-dose of cocoa administered to rats during the third trimester of gestation caused ductal constriction equivalent to that caused by indomethacin.^123^
- Normal human fetuses during the third trimester, when exposed to maternal consumption of polyphenols above the 75^th^ percentile of the average population, exhibit worse ductus arteriosus flow dynamics and increased right-to-left ventricular diameter ratios (higher ductal flow velocities and larger right ventricular diameters), in comparison with those exposed to maternal consumption of polyphenols below the 25^th^ percentile.^76^
- Normal human fetuses submitted to guided nutritional intervention (restriction of polyphenol-rich foods) in the third trimester showed, after 2 weeks, decreased ductal systolic and diastolic velocities, increased pulsatility index, and decreased right-to-left ventricular and pulmonary artery to aorta ratios, whereas these parameters did not change during the same period in control fetuses who were not submitted to the intervention.^77^
- Human fetuses with ductal constriction during the third trimester showed, in more than 95% of cases, reversion of the echocardiographic signs of this condition, as well as its hemodynamic compromise, after 3 weeks of a restricted in polyphenols diet, whereas there were no changes in the parameters evaluated in fetuses controls of the same gestational age, who did not receive a nutritional intervention with restricted maternal intake of polyphenols.^78^
- Polyphenol supplementation capsules inhibit physiological increase of PGE2 and other markers of inflammation and oxidative stress in women of childbearing age using combined hormonal contraceptives.^124^
- Dietary intervention to restrict maternal consumption of polyphenol-rich foods in the third trimester in cases of fetuses with ductal constriction is accompanied by an increase in plasma levels of PGE2, with improvements in the condition.^125^
- A 52-item food frequency questionnaire for quantifying consumption of polyphenol-rich foods in pregnant women, whose validity and reproducibility were evaluated in the South of Brazil, may be used in clinical practice.^71^

### 6.5. Conclusions

#### 6.5.1. Recommendations for Ductal Constriction Treatment

When ductal constriction is diagnosed in the fetal echocardiogram, the complete use interruption of NSAID should be recommended, in addition to the restriction of polyphenol-rich foods, made up of products with a concentration ≥ 30 mg/100 g of food, in accordance with the recommendations in table 6.2, intending to maintain balanced diet that includes all necessary micronutrients during this gestational period, reducing, however, the concentration of total polyphenols below 125 mg per day, or to the 25^th^ consumption percentile^78^ (Class of recommendation: I; level of evidence: A). If possible, consumption of other medications with potential anti-inflammatory actions (corticosteroids,^108^ aspirin,^107^ dipyrone,^105,110^ fluoxetine,^109,126^ paroxitine, sertraline,^109,111^ isoxsuprine,^107^ and naphazoline),^107^ their use interruption may be considered (Class of recommendation: IIa; level of evidence: C). In cases in which there is no reversal of ductal constriction and its consequences after initiation of treatment, preterm delivery may be considered, provided that fetal pulmonary maturity has been established (Class of recommendation: IIb; level of evidence: C).

**Table 6.2:** Recommendations for polyphenol-rich foods restriction after 28 weeks gestation for ductal constriction treatment

Restricted foods	Alternatives options
Raw beets: consume no more than 2 tablespoons/day	Cooked beets or carrots
Lettuce: consume no more than 10 medium-sized leaves/day	It is ideal to consume less
Red/purple plums, unpeeled: consume no more than 1 small unit/day	Pineapples, pears, and peeled red apples
Blackberries/mulberries: consume no more than 1/2 cup/day	Pineapples, acerolas, and limes
Red apples, unpeeled: do not eat the peel	Green apples or peeled red apples
Oranges/orange juice: do not consume	Pineapples, acerolas, limes, and tangerines*
Papaya: consume no more than 1 slice/day (formosa variety)	Guavas, acerolas, limes, and tangerines*
Strawberries: consume no more than 2 larges units/day	Pineapple, acerolas, limes, and tangerines*
Red/purple/pink grapes/grape juice: do not consume	White grapes, pears, and peeled apples
Green tea: do not consume	Fruit teas (teabags)
Black tea: do not consume	Fruit teas (teabags)
Boldo tea: do not consume	Fruit teas (teabags)
Coffee: do not consume	---
Yerba mate: do not consume	---
Dark/milk/bittersweet chocolate: do not consume	White chocolate
Cocoa powder: do not consume	
Olive oil: do not consume	Canola oil
Green herbs: consume no more than 12 teaspoons/day	Other natural spices

* Consume in moderation. When consuming a restricted food, consume no more than once daily, and do not exceed the quantities described in the table. Source: Adapted from Arnt et al.^127^

#### 6.5.2. Recommendations for Ductal Constriction Prevention

**Figure 6.1:**
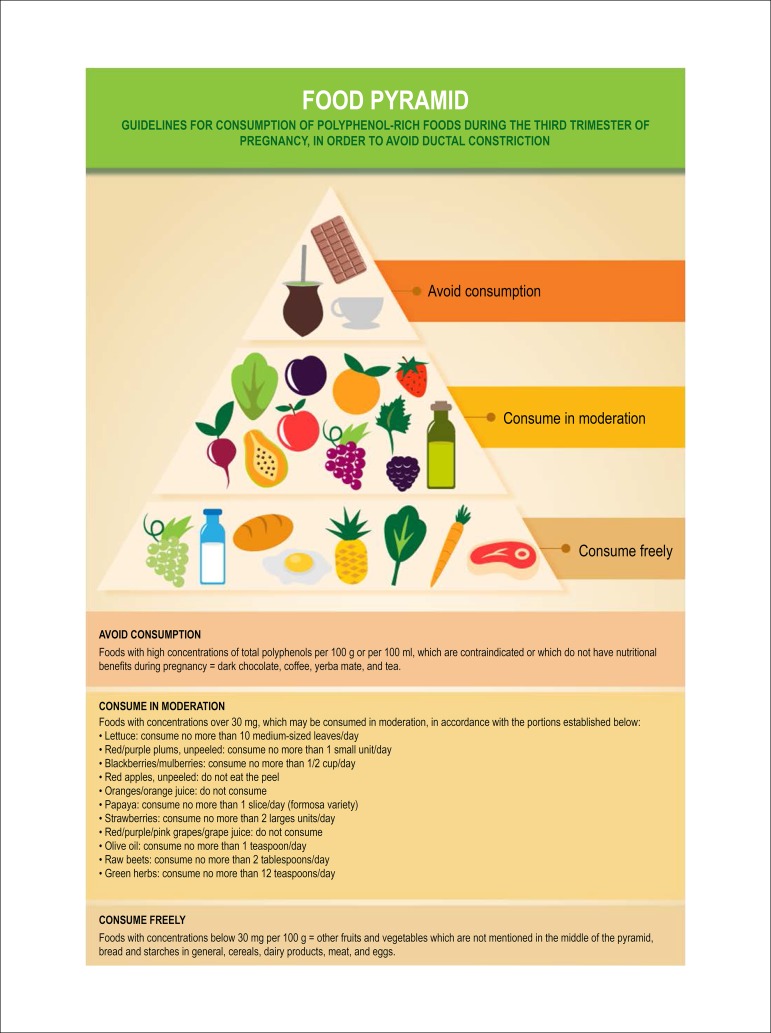
Food pyramid.

**Figure 6.2:**
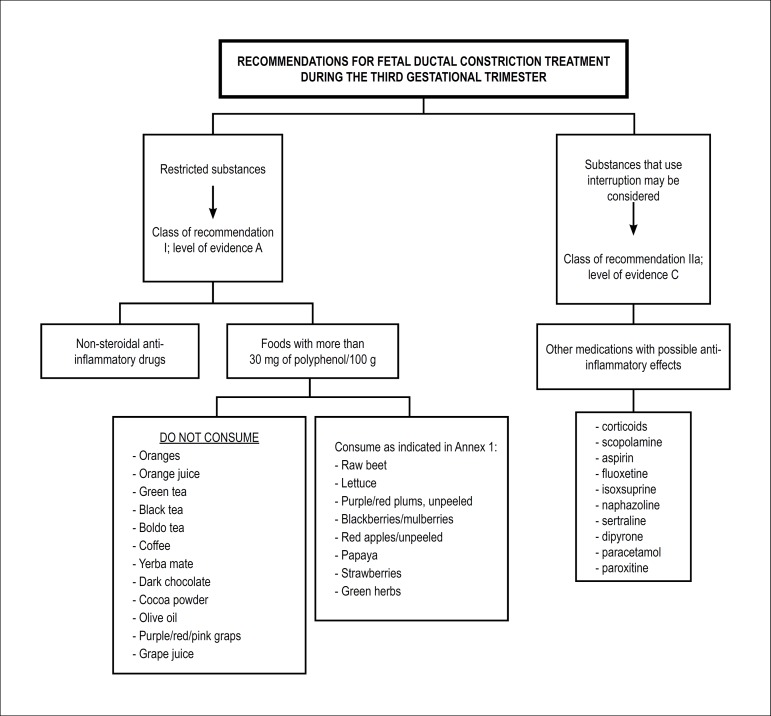
Recommendations for fetal ductal constriction treatment during the third gestational trimester. In cases in which there is no reversal of the ductal constriction and its consequences after initiation of treatment, preterm delivery may be considered, since fetal pulmonary maturity has been established. Class of recommendation: IIb; Level of evidence: C.

**Figure 6.3:**
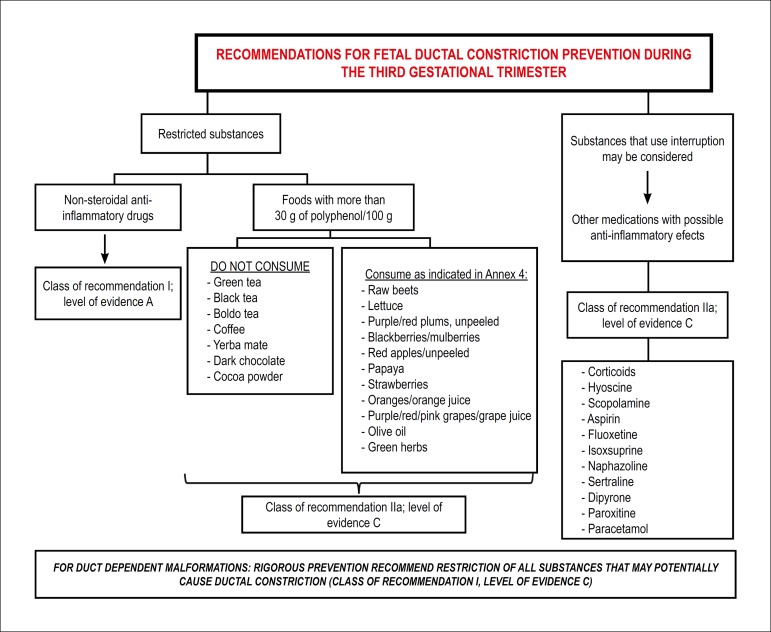
Recommendations for fetal ductal constriction prevention during the third gestational trimester.

In order to prevent fetal ductal constriction, pregnant women should be recommended not to use NSAID during the third trimester of gestation, regardless of the route of administration (Class of recommendation: I; level of evidence: A). It is also considered to recommend that they avoid using other medications with possible anti-inflammatory effects (corticosteroids,^108^ aspirin,^107^ dipyrone, fluoxetine,^109,126^ paroxetine, sertraline,^109,111^ isoxsuprine,^107^ and naphazoline)^107^ (Class of recommendation: IIa; level of evidence: C). It is sufficient to recommend moderate maternal polyphenol-rich foods consumption during the third trimester of gestation, i.e., below the 75^th^ percentile of consumption,^76^ or limiting consumption of foods with concentrations above 30 mg per 100 g, in accordance with the food pyramid shown in Figure 6.1. Reduced daily consumption of polyphenols below 1,089 mg (75^th^ percentile) maintains an acceptable diet for nutritional needs during this period of gestation (Class of recommendation: IIa; level of evidence: C). Figures 6.2 and 6.3, respectively, show recommendations for treatment and prevention of fetal ductal constriction.

## 7. Fetal Cardiac Arrhythmias: Diagnosis and Treatment

Screening programs for detecting prenatal cardiac abnormalities developed over the past 3 decades, improved the understanding of fetal cardiac rhythm abnormalities. Since fetal arrhythmias may severely compromise the gestation outcome, it is very important to diagnose, recognize the mechanisms, hemodynamic consequences, and the fetal cardiac morphology for perinatal therapy planning.

Fetal cardiac rhythm abnormalities affect approximately 0.5-2% of pregnancies, and are responsible for 10-20% of referrals for in utero cardiac examination. The ectopic beats are the most prevalent rhythm irregularities seen during fetal heart. They are usually benign however, may potentially trigger a sustained supraventricular tachycardia (SVT) especially when they are blocked. Some fetal cardiac arrhythmias, however, are considered emergencies in Fetal Cardiology, requiring early diagnosis and treatment and have determinant impact on perinatal morbidity and mortality. Complete heart block (CAVB), atrial flutter (AF), and SVT may have severe consequences for the fetus clinical status.^128^

### 7.1. Fetal Cardiac Rhythm and Fetal Cardiac Arrhythmias

During fetal echocardiography, heart rate and rhythm are assessed with simultaneous examination of the atrial and ventricular systoles employing M-mode, two-dimensional echocardiography and pulsed-wave Doppler with or without color flow mapping. Cardiac rhythm is considered normal when the ratio of atrial and ventricular contractions is 1:1, with heart rate ranging from 120 to 180 bpm.^48,129-131^

M-mode allows to evaluate the movement of the posterior atrial wall (atrial systole or A wave), concomitantly with aortic valve opening (ventricular systole or V wave). This trace is obtained from the longitudinal two-dimensional image of the heart, with the cursor positioned crossing the right ventricle, the aortic valve and the LA. Sinus rhythm is identified when, for each movement of the left atrial wall (A wave), there is a corresponding opening movement of the aortic valve (V wave), i.e., 1:1 atrioventricular (A:V) conduction. Positioning the cursor simultaneously across the atrial (A wave) and ventricular (V wave) wall may also be employed. Color M-mode facilitates the identification of aortic flow during ventricular systole and may also be used to identify left atrial activity from mitral flow.

The atrioventricular sequence may also be assessed positioning the pulsed Doppler sample volume between the left ventricular inflow and outflow tracts, thus recording the mitral (A wave) and aortic (V wave) flows. Additionally, the sample volume may be placed between the SVC and the aorta in the 3 vessels view. The SVC "A" wave reversal flow represents the atrial contraction (A wave), and the aortic flow represents ventricular systole (V wave). The same concept can be used with the Doppler sample volume placed at the same time reaching the pulmonary artery and vein flows.^48,129-138^

### 7.2. Extrasystoles

Extrasystoles occur in 1-3% of pregnancies. They are, usually, benign, with no consequences for the fetus. In the setting of bigeminy, trigeminy, or very frequent extrasystoles (1 for every 3-5 fetal heartbeats), differential diagnosis with ventricular extrasystoles, long QT syndrome, and second-degree atrioventricular block may be difficult. The presence of blocked bigeminy increases the risk of SVT triggering.^139-141^

#### 7.2.1. Isolated Supraventricular Extrasystoles

Correspond to premature atrial contractions (A wave), that may or may not be followed by ventricular activity (conducted or blocked, respectively). They may occur with bi- or trigeminy, compensatory pauses, or in series. They are considered benign arrhythmias, and do not require treatment. About 1% of conducted ectopic beats may trigger tachyarrhythmias.^48^

#### 7.2.2. Ventricular Extrasystoles

Ventricular extrassystoles are ventricular ectopic beats that are not related to atrial activity.

Table 7.1 shows the summary of in utero management of irregular rhythms.

**Table 7.1:** In utero management of irregular rhythm

Diagnosis	Cause	In utero management	GOR/LOE	Comments
Second-degree AVB	Autoimmune	Dexamethasone	IIb/B	This may stop progression to CAVB
	Structural CHD	Weekly follow-up	I/C	If possible, perform FMCG to rule out LQTS
	Channelopathy	Weekly follow-up	I/C	
VPC or frequent APC	Idiopathic	Observation with obstetric evaluation of fetal HR weekly until the arrhythmia is resolved (bigeminy, trigeminy, or 1 ES at every 35 beats)	I/A	2% also have first- or second-degree AVB
	Oval fossa aneurysm			For APC, there is a 0.51% risk of developing SVT
				For VPC, the risk of developing VT is unknown
				Most episodes are benign and of short duration
				Evaluate secondary causes
Secondary causes				
VPC or frequent APC	Myocarditis	Observation with evaluation of FHR at weekly intervals	I/C	
		Frequent evaluation (every 12 week) of heart function and other parameters of fetal CHF		
	Cardiac tumors	Observation with obstetric evaluation of FHR weekly	I/C	
	Ventricular or atrial diverticula or aneurysm	Observation with FHR assessment by OB weekly	I/C	
	Maternal stimulants	Observation with FHR assessment by OB	I/C	

APC: atrial premature contractions; AVB: atrioventricular block; CAVB: complete atrioventricular block; CHD: congenital heart disease; CHF: congestive heart failure; FHR: fetal heart rate; FMCG: fetal magnetocardiography; GOR: grade of recommendation; LOE: level of evidence; LQTS: long QT syndrome; SVT: supraventricular tachycardia; VPC: ventricular premature contraction; VT: ventricular tachycardia. Source: adapted from Donofrio et al.^17^

### 7.3. Fetal Bradycardia

Fetal bradycardia is considered when the fetal heart rate of < 110 bpm. When treatment is necessary, it is important to identify its cause and mechanism.

#### 7.3.1. Sinus Bradycardia

Sinus bradycardia is diagnosed when the heart rate is < 110 bpm with a 1:1 A:V conduction. It is usually a vagal response secondary to hypoxia or umbilical cord compression by the transducer. It also may occur due to maternal illnesses. When transitory, they are commonly benign and do not require treatment. However, persistent bradycardia indicates fetal abnormality and its causes should be treated.^48,129,134,137,141^

#### 7.3.2. Low Atrial Rhythm

The main mechanisms of low atrial rhythm include congenital displacement of atrial activation, acquired damage of the sinoatrial node, channelopathy, and secondary suppression of sinus node rate. Left and right atrial isomerism can occur, with fetal heart rate varying from 80 to 130 bpm. Situations that may cause sinus node fibrosis, such as maternal anti-Ro/anti-LA antibodies or viral myocarditis, may occur with progression to fetal death. Additionally, maternal use of medications, such as sedatives or betablockers, may reduce the sinus node rate. Low atrial rhythm does not require treatment.^137^

#### 7.3.3. Blocked Atrial Bigeminy

Blocked atrial bigeminy occur with a heart rate ranging from 75 to110 bpm in a 2:1 atrioventricular conduction. They do not require treatment. It is known, however, that approximately 10-13% may evolve to SVT; weekly evaluation of fetal heartbeats is thus recommended by echocardiogram or sonar.^137,142^

#### 7.3.4. Complete Atrioventricular Block

CAVB results in complete dissociation between atrial and ventricular activity, with heart rates usually below 60 bpm. In 50-55% of cases, malformation of the conduction system occurs, as a consequence of structural heart diseases, such as congenitally corrected transposition of great arteries and left atrial isomerism.^141,143-146^ In about 40% of the cases, it occurs due to maternal autoimmune diseases that present with anti-SSA/SSB (anti-Ro/LA antibodies).^142-147^

The risk increases in the presence of anti-Ro 52-kd (sequence p200) antibodies, that cannot be tested in Brazil yet.^147-153^ In a minority of cases, no etiology is identified. Fetuses without hydrops and with heart rate above 55 bpm have good prognoses. In immature fetuses, with very early hydrops and heart rates below 50 bpm, prognosis is more limited. Fetuses with CAVB and structural heart diseases, such as left atrial isomerism, have a poor prognoses.^145^

**Figure 7.1:**
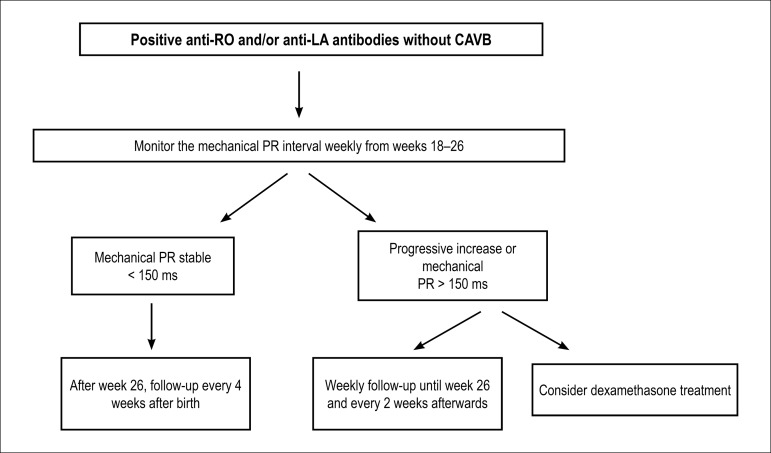
Suggested approach for pregnant women with positive antibodies, without fetal CAVB. CAVB: complete atrioventricular block; ms: miliseconds.

In mothers with autoimmune diseases, it is recommended to test maternal anti-SSA/RO antibodies. If positive, and the fetus is in sinus heart rhythm, weekly measurements of the AV interval (mechanical PR interval) are recommended, from weeks 18 to 26. This measurement should be taken employing pulsed-wave Doppler, evaluating mitral and aortic flows simultaneously, from the beginning of the mitral A wave ("A") to the beginning of the ventricular systole ("V").^142^ Myocardial function should be monitored every 4 weeks up to delivery (grade of recommendation: I; level of evidence: C) (Figure 7.1).^154^

Although controversial, treatment with dexamethasone at a dose of 4-8 mg orally can be started to cases where the AV interval is > 150 milliseconds or when it increases progressively. Some groups have shown to be beneficial the treatment of immune CAVB with maternal dexamethasone (4-8 mg orally) and/or intravenous gammaglobulin infusion,^149-158^ observing reduced inflammatory response, stabilization of first- and second-degree AVB, regression of endocardial fibroelastosis and hydrops improvement.^145-151^

However, the use of corticosteroids may be associated with complications, such as ductal constriction, maternal diabetes, restricted growth, and oligohydramnios.^149-161^

Dexamethasone may be used to treat first- and second-degree AVB associated with signs of myocardial inflammation (myocardial hyperechogenicity, valve regurgitation, cardiac dysfunction, and pericardial effusion) to prevent progressing to CAVB, however the efficiency of corticosteroids has not been completely established and one may consider its possible side effects.^140^ In fetuses with CAVB without functional consequences, dexamethasone may also be used to reduce the prevalence of dilated cardiomyopathy.^152,162^ Whenever significant side effects occur in the mother or the fetus, the use of the medication should be interrupted. Intravenous immunoglobulin associated with dexamethasone may improve survival in fetuses with endocardial fibroelastosis or systolic dysfunction.^149^ It is not yet known, however, when is the ideal moment for administration and the ideal intervals between doses. There is no recommendation regarding the prophylactic use of immunoglobulin at the beginning of gestation for mothers with positive antibodies.^160^

The use of salbutamol, terbutaline, or isoprenaline is indicated when heart rate is < 55 bpm and/or in the presence of fetal heart failure and hydrops.^142,146,157^ These medications are usually well tolerated. Maternal extrasystole and sinus tachycardia may appear.^161^ There is an increase in fetal heart rate of approximately 10 to 15% of the basal frequency, and, although small, it may prolong the gestation to or close to term. There are no studies demonstrating that these medications modify fetal survival in these cases. In immature fetuses with hydrops, with very low heart rate, in utero implantation of a pacemaker may be considered. This procedure continues to have technical limitations and is still undergoing experimental studies.

**Figure 7.2:**
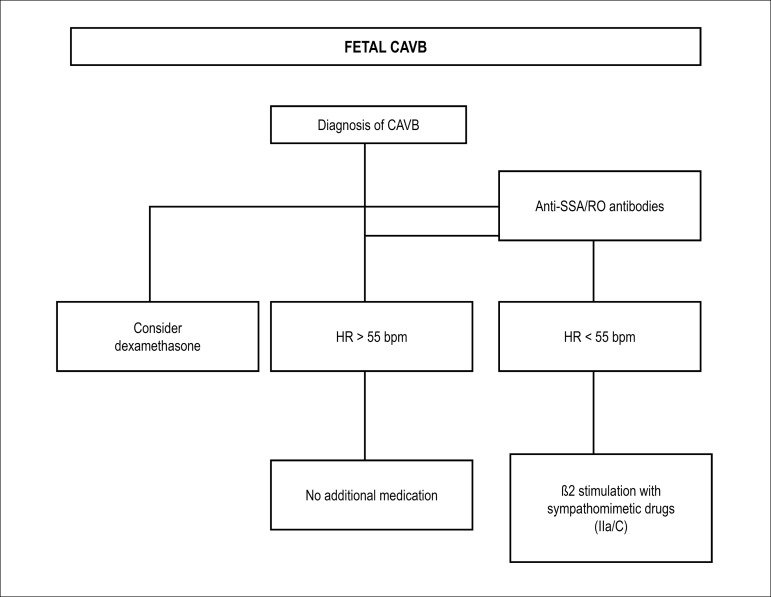
Suggested approach for fetuses who have CAVB. CAVB: complete atrioventricular block; HR: heart rate.

Indications for delivery should be analyzed based on the degree of fetal manifestations. In fetuses with significant hydrops, with ventricular rate < 50 bpm and pulmonary maturity (after week 34 of gestation), delivery should be considered, with immediate postnatal pacemaker implant.^163^ In fetuses before week 26 of gestation, with heart rate < 45 bpm and hydrops, in utero pacemaker implant, still in experimental phase, may be a therapeutic option.^164-168^ In fetuses between weeks 26 and 34 of gestation, the risks of prematurity and the manifestations of CAVB should be weighed together. The in utero suggested manaegment of fetal bradycardia is summarized in figure 7.2.

### 7.4. Fetal Tachycardia

Fetal tachycardia is diagnosed when fetal heart rate is > 180 bpm. In utero treatment depends on gestational age, etiology, degree of hemodynamic compromise (presence of hydrops), mother's clinical condition, and potential maternal risks of fetal treatment. The therapeutic decision should be based on fetal vs. maternal risks. Medical treatment is indicated for fetuses with sustained or intermittent tachycardias with hydrops and/or ventricular dysfunction, unless gestation is close to term, with fetal pulmonary maturity, thus minimizing the risks of preterm birth.^17,129,131-133,136,140,142,169,170^

**Figure 7.3:**
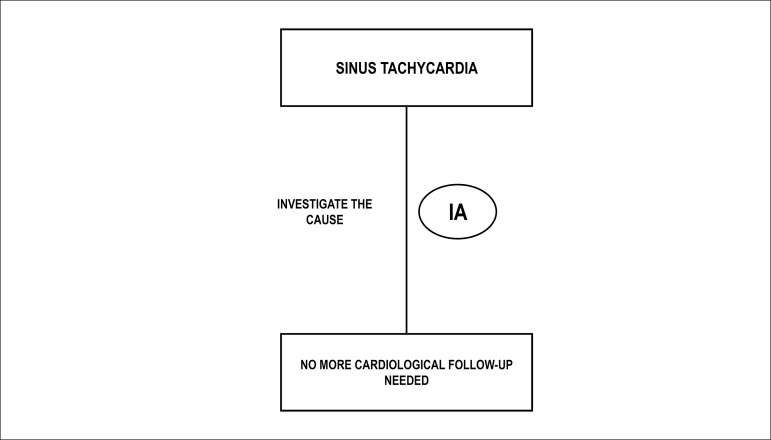
Sinus tachycardia clinical management.

**Figure 7.4:**
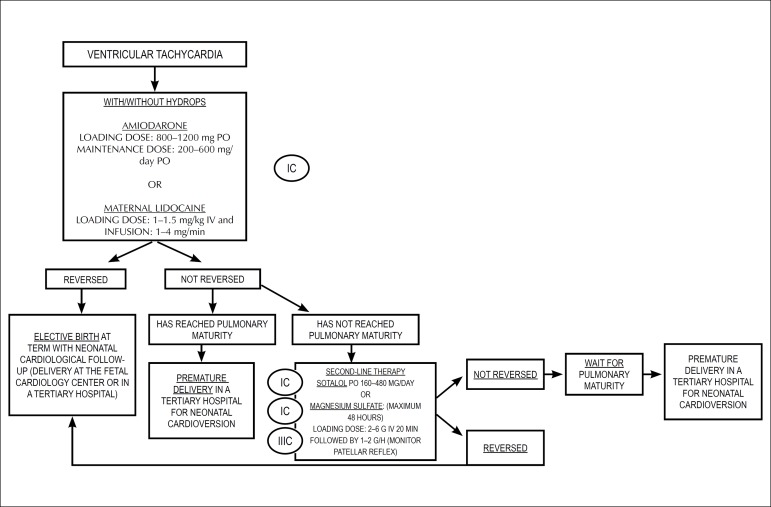
Treatment flowchart for ventricular tachycardia. IV: intravenous; PO: orally.

**Figure 7.5:**
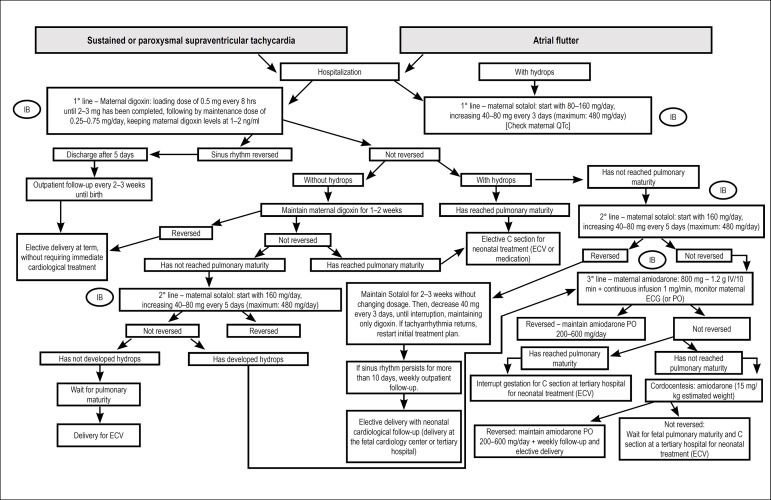
Flowchart treatment for supraventricular tachycardias. ECV: electric cardioversion; IV: intravenous; PO: orally.

Tables 7.3 and 7.4, respectively, demonstrate the management of tachyarrhythmias and antiarrhythmic drugs.^17^ The suggested management approaches for fetal tachycardias are shown in Figures 7.3, 7.4, and 7.5.

**Table 7.3:** In utero management of tachycardias

Diagnosis	In utero management	GOR/LOE	Comments
**Intermittent tachycardia**			
SVT or AF	Observation	I/B	Frequent fetal HR auscultation
VT ≥ 200 bpm	Antiarrhythmic medication	IIa/C	
**Sustained tachycardia**			
SVT or AF with hydrops or ventricular dysfunction	First or second line (transplacental) drugs:		See Table 7.4, for dosing ranges and monitoring recommendations
	Digoxin	I/B	
	Sotalol	I/B	
	Combination of drugs (transplacental)	IIb/B	Combination treatments are used for severe, drug-refractory cases. Consider preterm delivery if near term
	Third line (transplacental):		
	Amiodarone	I/B	
	Contraindicated: verapamil	III/A	
	Contraindicated: procainamide	III/B	
	Direct fetal treatment:		
	IM digoxin	IIa/B	
	Intracordal digoxin	IIb/B	
	Contraindicated: Intracordal adenosine	III/B	
SVT ≥ 200 bpm, without hydrops or ventricular dysfunction (usually SVT has HR ≥ 220 bpm; consider other causes if HR < 220 bpm).	First or second line:		
	Digoxin	I/B	See Table 7.4, for doses and monitoring recommendations
	Sotalol	I/B	Frequent monitoring of fetal well-being and maternal/fetal drug toxicity. Consider preterm delivery if near term.
	Third line:		
	Amiodarone	IIb/B	
	Contraindicated: verapamil	IIb/A	
	Contraindicated: procainamide	III/B	
	Observation	I/B	
SVT < 200 bpm, without hydrops or ventricular dysfunction	Sotalol	I/B	Digoxin increases AVB and decreases ventricular response. Consider preterm delivery if near term
AF	Digoxin	I/B	
	Amiodarone	IIb/B	
	Contraindicated: procainamide	III/B	
VT ± hydrops		I/C	
First line treatment	Magnesium IV Lidocaine IV Propranolol (oral)	I/C	FMCG (if available) to measure QTc interval. Start with magnesium IV, then lidocaine, load + maintenance. Note: maternal intravenous magnesium should not be used for > 48 h. Consider preterm delivery if near term.
Second line treatment	Mexiletina (oral) Sotalol	I/C	

AF: atrial flutter; GOR: grade of recommendation; IV: intravenous; HR: heart rate; IM: intramuscular; FMCG: fetal magnetocardiography; LOE: level of evidence; SVT: supraventricular tachycardia; VT: ventricular tachycardia.

Source: adapted from Donofrio MT et al.^17^

**Table 7.4:** Antiarrhythmic drugs

Drug	Therapeutic dose	Therapeutic serum level and effect	Toxicity
Digoxin	LD: 0.5 mg (2 capsules) every 8 h for 48 h 1.5 mg/d for 2 days	0.7-2.0 ng/mL	Maternal nausea/vomiting, sinus bradyarrhythmia or AVB, proarrhythmia
	MD: 0.250.75 mg/day Fetal IM dose: 88 µg/kg every 12 h, repeat twice	Nausea, fatigue, loss of appetite, sinus bradycardia, first-degree AV block, nocturnal Wenckebach AV block (rare)	Fetal IM: sciatic nerve injury or skin laceration from injection
Sotalol	160480 mg/day every 812 h PO	Levels not monitored	Nausea/vomiting, dizziness, QTc ≥ 0.48 s, fatigue, BBB, maternal/fetal proarrhythmia
		Bradycardia, first-degree AVB, P and QRS widening, QTc ≤ 0.48 s	
Amiodarone	LD: 18002400 mg/d divided every 6 h PO	0.72.0 µg/mL	Nausea/vomiting, thyroid dysfunction, photosensitivity rash, thrombocytopenia, BBB, QTc ≥ 0.48 s, maternal/fetal proarrhythmia, fetal torsades with LQTS, fetal goiter, neurodevelopmental concerns
	MD: 200600 mg/d PO	Maternal/fetal sinus bradycardia, decreased appetite, first-degree AVB, P and QRS widening, QTc ≤ 0.48 s	
	Consider discontinuation of drug and transition to another agent once normal rhythm is reestablished or hydrops has resolved.		
Propranolol	60320 mg/d divided every 6 h PO	25-140 ng/mL	Fatigue, bradycardia, hypotension, AV block, fetal growth restriction, increased uterine tone
		First-degree AVB, bradycardia, increased uterine tone	
Lidocaine	LD: 11.5 mg/kg followed by infusion of 14 mg/min continuous IV	1.5-5 µg/mL	Nausea/vomiting, neurological symptoms, proarrhythmia
Mexiletine	600900 mg/d divided every 8 h PO	0.5-2 µg/mL	Nausea/vomiting, neurological symptoms, proarrhythmia
Magnesium sulfate	LD: 26 g IV over 20 min followed by 12 g/h	< 6 mEq/L	Fatigue, neurological symptoms If there is loss of patellar reflex and/or levels of> 6 mEq/L STOP infusion
	Treatment for > 48 h is not recommended but redosing may be considered if VT recurs	Monitor patellar reflex	Levels > 5 mEq/L associated with maternal changes on ECG and proarrhythmia

AV: atrioventricular; AVB: atrioventricular block; BBB: bundle-branch block; ECG: electrocardiogram; IM: intramuscular; IV: intravenous; LD: loading dose; LOE: level of evidence; LQTS: long QT syndrome; MD: maintenance dose; PO: orally; VT: ventricular tachycardia.

Source: afapted from Donofrio et al.^17^

#### 7.4.1. Intermittent Tachycardias

Intermittent tachycardia is defined when it is present for less than 50% of the exam period, the minimum observation time being 30 minutes. Sinus tachycardia is determined by atrial and ventricular activation with 1:1 A:V conduction and heart rate over 160 bpm and, usually, below 180 bpm. It is frequently associated with an underlying fetal or maternal abnormal condition, such as fever, stress, or use of medication. Its cause should be treated. As an isolated finding, it does not have clinical significance and does not require treatment.^137,138,141^

Intermittent ventricular tachycardia, with ventricular rate over 200 bpm is extremely rare and may evolve to important hemodynamic impairment and hydrops; for this reason, treatment is indicated.

Other intermittent tachycardias usually do not have signs of cardiac hemodynamic impairment, and there is no indication for treatment.^171^ However, in isolated cases, it may evolve to sustained tachycardia, justifying its follow up.

#### 7.4.2. Sustained Tachycardias

This group of fetal arrhythmias, identified by a period of more than 50% of exam duration, includes supraventricular tachycardias, AF, and ventricular tachycardias. The therapeutic goal is to bring the gestation to term, while improving secondary manifestations. Prognosis is good when they are reversed in utero and limited for immature fetuses with hydrops and cases of arrhythmia which were not successfully reversed. Prognosis should be considered favorable when fetuses continue with tachyarrhythmia, but with lower heart rates and improvement in hydrops.

##### 7.4.2.1. Diagnosis

Sustained atrial tachycardia is characterized by a cardiac rhythm with 1:1 A:V conduction and heart rate above 180 bpm, usually above 220 bpm.^17,137,138,141^ It is important to understand the underlying mechanism of the arrhythmia, assessing simultaneously the atrial and ventricular activity. Using Doppler flow tracing, it is possible to measure the AV (atrium ? ventricle) and VA (ventricle < atrium) intervals, which correspond, analogously and respectively, to PR and RP intervals in an electrocardiogram. When the VA interval is greater than the AV, the most possible diagnosis is reentrant tachycardia (95%); when the VA interval is greater than the AV, tachycardia due to ectopic atrial focus or junctional reciprocating tachycardia are the most frequent diagnosis.^132,133,136^

Atrial flutter presents with atrial rates above 400 bpm, with variable atrioventricular conduction (2:1, 3:1, 4:1) and ventricular rates (200-250 bpm).^17,137,138,141,169^

Ventricular tachycardia is identified as atrioventricular dissociation, with atrial rate lower than ventricular, varying from 100 to 400 bpm. When it coexists with bradycardia periods, the possible diagnosis is long QT syndrome, which may manifest as monomorphic ventricular tachycardia, torsade de pointes, ventricular dysfunction, atrioventricular valve regurgitation, and fetal hydrops.^138,172^

##### 7.4.2.2. Treatment

The first choice for medical treatment of supraventricular tachycardias in most centers continues to be transplacental digoxin, given that it is safe and widely used during gestation.^17,137,138,141,173,174^ The doses should be high, since only 50-70% crosses the placental barrier. The recommended loading dose is 3.0 mg during the first 48 hours of treatment, i.e., 0.50 mg every 8 hours. The maintenance dose is 0.25-0.75 mg/day, varying in accordance with isolated experience of each service and maternal serum level. Daily control of digoxin level is mandatory, and it should be kept between 1 and 2 ng/mL. If it is not possible to administer it orally, intravenous lanatoside C may be used as an alternative. If the arrhythmia has not reversed after 5 days, oral sotalol is initiated as second-choice drug.^175-177^ This may be used with an initial dose of 80 mg every 12 hours, gradually increasing 40-80 mg every 3-5 day, until the arrhythmia is reversed or the maximum dose of 480 mg/day has been reached. In this case, the mother must remain in hospital for monitorization, with daily ECG control to measure the QTc interval, as well as serum levels of digoxin. In fetuses with significant hydrops and sustained tachycardia with elevated heart rate, sotalol may be initiated concomitantly with digoxin. Combined therapy has greater risks of maternal and fetal complications. If there is no therapeutic response in fetuses who are severely affected, the third-choice drug, amiodarone, may be used at a dose of 800-1,200 mg/day.^178-179^ This drug, however, has a significant toxicity for both mother and fetus.^180^

If the fetal tachyarrhythmia continues, with important hemodynamic impairment and severe hydrops, direct fetal therapy may be necessary, via cordocentesis or direct intramuscular injection, given that, in this situation, there is a significant decrease in the transplacental passage of medications.^171,181,182^ The risks and benefits of every situation must be weighed individually. Digitalis (dose of 0.03 mg/kg) or amiodarone (dose of 15 mg/kg) may be administered. Adenosine has not shown any effect in maintaining sinus rhythm, and it is not recommended for atrial flutter.

Some centers use flecainide (not commercially available in Brazil) or sotalol as first-choice drugs.^130,177,183,184^ After birth, treatment should be based on the therapy used in utero and on the tachycardia mechanism. It is recommended to maintain therapy for 6 months to 1 year, in accordance with the outcome. About 50% of cases of fetal SVT do not recur after birth.^185^

For AF, recommended medication for initial treatment may be either digoxin and/or sotalol.^185^ Sotalol is also safe and efficient with a reversal rate to sinus rhythm of 50-80%, without mortality.^177^

Doses and forms of administration for AF are as previously described for SVT. Following delivery, synchronized cardioversion is indicated when there is no in utero reversal. After birth, AF does not usually recur once it has been reversed and maintenance of medical treatment is not recommended.

Sustained ventricular tachycardia with ventricular rate < 200 bpm is usually well tolerated; when it exceeds this rate, transplacental magnesium is recommended. This infusion should not be administered for more than 48 hours.^172,186,187^ If ventricular tachycardia recurs, a new dose of magnesium may be used, provided that maternal serum levels are < 6 mEq/L and there are no signs of toxicity. Therapeutic options for pharmacological treatment of sustained ventricular tachycardias include oral administration of amiodarone, propranolol, and mexiletine or maternal intravenous lidocaine. Amiodarone, sotalol, and flecainide cannot be used when there is long QT syndrome.^186,187^ When ventricular tachycardia is secondary to myocarditis or maternal antibodies, intravenous dexamethasone and immunoglobulin may be administered to the mother. This treatment should be continued after birth.

One should consider that treatment of fetal sustained tachycardias is slow and its goal is to bring gestation to term. Total reversal of arrhythmia and hydrops may occur several weeks after initiation of medical treatment. A concomitant Doppler ultrasound, performed by the obstetrician, is essential to decide if the delivery should be anticipated. Delivery anticipation should be limited to fetuses with imminent risk of in utero death. If sustained tachycardia persists in fetuses with severe hydrops and proven pulmonary maturity (after week 34 of gestation), it is recommendable to deliver the baby and begin postnatal treatment immediately.

## 8. Fetal Cardiac Interventions

The potential benefits of fetal cardiac interventions have been emphasized for many years. In the year 2000, Kohl et al.^188^ published the worldwide experience of fetal aortic valvuloplasty, which, at that time, consisted of 12 cases, with 7 technically well-succeeded but only 1 survival. Since that time, the Boston Children's Hospital group has initiated an invasive intrauterine cardiac therapy program, stimulating vast progress in the field and disseminating technical application throughout various other centers around the world.^189,190^

The main reason for invasive procedures during fetal life is to improve outcome and postnatal prognosis, either because the fetus is at a risk of not surviving or because postnatal outcome is strongly unfavorable. Early therapy for CHD may improve the chances of myocardial and vascular remodeling and offer better chances of adapting the blood supply to the developing myocardium. Thus, provided that the technique is well established, the equipment is appropriate, and, above all, the medical team is trained in fetal surgery, pediatric interventions, and Fetal Cardiology, fetal percutaneous interventions represent another form of therapy in the field of Pediatric Cardiology.^189^

The main heart diseases that benefit from intervention in utero are HLHS with severe flow restriction through the interatrial septum, critical aortic valve stenosis with impending left ventricular hypoplasia, and pulmonary atresia with intact interventricular septum (PAIVS), or critical pulmonary stenosis with right ventricular hypoplasia.^191^

### 8.1. Indications

The main indications for fetal cardiac interventions are summarized in Table 8.1 and subsequently described.

**Table 8.1:** Main indications for fetal cardiac interventions

**Aortic valvuloplasty**	Gestational age between 22 and 30 weeks
**Critical aortic stenosis with impending HLHS**	Thick aortic valve with little mobility
	Minimal or no aortic anterograde flow
	Reverse flow in the transverse arch
	Reverse shunt at the atrial level (L®R)
	Monophasic LV inflow (single E wave of short duration)
	Moderate or severe LV systolic dysfunction (subjective analysis)
**Critical aortic stenosis with giant LA**	Same criteria as previously described
	LV function may not be very abnormal due to the presence of massive mitral regurgitation
	Giant LA
**Pulmonary valvuloplasty**	Gestational age between 22 and 30 weeks
**Pulmonary atresia with intact interventricular septum/critical pulmonary stenosis**	Thick pulmonary valve with little or no mobility
	Minimal or no pulmonary anterograde flow
	Inverted flow in the ductus arteriosus, i.e., aorta®pulmonary
	Monophasic RV inflow (single E wave of short duration)
	Some degree of RV hypoplasia or no growth during 24 weeks of observation
**Balloon atrial septostomy**	Gestational age between 28 and 33 weeks
**HLHS or variants with intact interatrial septum or minimal foramen ovale**	Minimal or no flow at the atrial level
	Dilated LA and pulmonic veins
	Biphasic and bidirectional pulmonary vein Doppler tracing

HLHS: hypoplastic left heart syndrome; L: left; LA: left atrium; LV: left ventricle; R: right; RV: right ventricle.

#### 8.1.1. Critical Aortic Stenosis with Impending Hypoplastic Left Heart Syndrome

Aortic stenosis is defined as the following morphological and functional characteristics: thick valve, little mobility, and turbulent or no antegrade flow across the valve assessed by Doppler techniques. Left ventricular to aorta Doppler gradient should not be used to classify the severity of the stenosis since, there is a frequent association of endocardial fibroelastosis and severe myocardial dysfunction in critical aortic stenosis. Reverse flow in the transverse arch, i.e., coming from the descending aorta to the ascending aorta; inverted flow at the atrial level (from left to right); monophasic left ventricular inflow (Doppler tracing across the mitral valve showing single A wave due to high filling pressures), and moderate or severe left ventricular dysfunction are the main functional parameters that suggest impending HLHS.^192-195^ Ideally, when fetal intervention is considered to avoid left heart hypoplasia, left ventricular length Z score (long axis) should be > −2, meaning that the left ventricle is not hypoplastic yet. Occasionally, aortic valvuloplasty is performed in cases where the left ventricle has already some degree of hypoplasia (Z-score > −4 and < −2), and the main aim in these cases is to promote some anterograde aortic flow, which may improve coronary and encephalic perfusion and allow ascending aorta growth, knowing that the chances of left ventricular complete recovery are low.^192-195^

#### 8.1.2. Hypoplastic Left Heart Syndrome with Intact Interatrial Septum or Significantly Restrictive Foramen Ovale

This situation is characterized by absent or minimal high velocity flow across the interatrial septum and bidirectional flow in the pulmonary vein with prominent reverse flow, with disappearance of the classic triphasic pattern.^196,197^

#### 8.1.3. Pulmonary Atresia with Intact Interventricular Septum or Critical Pulmonary Valve Stenosis with Signs of Evolving Right Heart Hypoplasia

This disease is defined as membranous pulmonary atresia with identifiable pulmonary valve leaflets with intact interventricular septum, associated with minimal or no anterograde pulmonary blood flow; reverse flow in the ductus arteriosus, i.e., coming from the aorta to the pulmonary artery; some degree of right heart hypoplasia, with hypoplastic tricuspid valve annular diameter (Z score < −2), or evidence that the right ventricle has not grown during 2-4 weeks of observation. Cases with significant coronary to right ventricle fistulas are excluded.^198-200^

#### 8.1.4. Critical Aortic Stenosis with Massive Mitral Regurgitation and Giant Left Atrium

This is a specific group of fetuses that has only recently been characterized as a subgroup of critical aortic stenosis. These cases present with left ventricular dilation, reverse flow in the transverse arch, and some degree of left ventricular dysfunction. Most of them are associated with fetal hydrops and may benefit from aortic valvuloplasty associated or not with atrial septostomy to reduce the risk of fetal or neonatal death.^44,201^

### 8.2. Technical Considerations

Pre-anesthesia fasting and tocolytic prophylaxis consist the main preparation for the procedure. Nifidipine, 20 mg orally, started 4-8 hours before the procedure, is the medication of choice for this purpose, since it has few side effects and is highly effective.^189^ The intervention is performed under maternal regional block, preferably via spinal anesthesia. General anesthesia may also be used, but this has the disadvantage of hindering proper fetal positioning, given that maternal general anesthesia also anesthetizes the fetus.

The fetal positioning is obtained with manual maneuvers allowing the fetal specialist to reach the target cardiac structure percutaneously. The ideal fetal position is pelvic with the spine downwards, leading to proceed the puncture as close as possible to the uterine fundus.^189^

Fetal anesthesia may be intramuscular or intravenous via the umbilical cord. It is performed with a mixture of opioid (fentanyl), muscle blocker (pancuronium), and atropine at doses of 15 µg, 0.2 mg, and 0.02 mg per kilogram of fetal weight, respectively. A 20-G Chiba needle is used to administer this medication.^189,190,194^

The heart is also accessed with a Chiba needle, 15 cm in length ranging from 17 to 19 Gauge. The entire procedure is monitored by ultrasound, which may be operated by either the fetal specialist or the fetal cardiologist. Once the abdominal wall is crossed, the needle reaches the amniotic cavity and the fetal thorax.^194^ The target structure (aortic valve, pulmonary valve, or interatrial septum) is reached by direct heart puncture.

Once the distal end of the needle has reached the target cardiac structure, a pre-assembled coronary angioplasty balloon catheter is advanced through the needle until the balloon is positioned across the structure to be dilated. The pressure with which the balloon is inflated varies, considering the diameter and the target structure. For semilunar valve dilation, the ideal balloon:annulus ratio is from 1.1 to 1.2.^193^ After the balloon is completely deflated, the entire set (balloon, catheter, and puncture needle) is removed all together, at once. After the system is complete removed from the fetal heart, bradycardia and hemopericardium frequently occur.^190^ Voluminous effusions should promptly be emptied via a new puncture with a 20-G needle.^194^ Removal of 1-2 ml of blood from the pericardium is usually enough to treat the condition. In most cases, this does not cause fetal anemia.

These procedures are not exempt from risks involving the mother and/or the fetus. Maternal risks are currently extremely low and minimized, thanks to the increased experience in fetal surgery for noncardiac diseases. These complications include premature rupture of membranes, infection, hemorrhage, placental abruption, preterm labor, anemia, bradycardia, and fetal death.^202^

There are still some doubts regarding the ideal moment to perform fetal cardiac intervention. Due to the reduced number of candidates and the morphological variability that every pathology may present, it is difficult to establish when it should be considered too late for intervention.^203^ It seems reasonable to perform intervention as early as possible, soon after the diagnosis. From the technical point of view, however, it is very difficult to act before gestational week 20, due to the small dimensions of the fetal heart. Interventions performed very early may result in orifice and valve closure before the fetus has reached term.^203^ On the other hand, late interventions do not prevent ventricular hypoplasia or avoid vascular damage of the pulmonary circulation. It appears to be consensual that the adequate period would be between gestational weeks 22 and 30.^190^

### 8.3. Aortic Valvuloplasty

The goal of aortic valvuloplasty is to change the natural history of critical aortic stenosis, maintaining left ventricular size and function adequate for biventricular physiology at birth or after a rehabilitation process. Alleviating left ventricular outflow obstruction reduces the left ventricular myocardial damage, thus facilitating chamber growth and myocardial function improvement. This hypothesis is based on animal models studies, which demonstrated the impact of load and flow conditions abnormalities on the developing myocardium, which leads to abnormal cardiovascular growth and function conditions.^204-209^ According to the study published by McElhinney et al.,^195^ there are anatomical and functional characteristics that are predictive of technical success and progression to postnatal biventricular circulation, based on the experience of 70 fetal aortic valvuloplasty procedures performed by their group.^8^ These criteria are shown in Table 8.2.

**Table 8.2:** Criteria for technical success (initial criteria) and criteria that indicate potential outcome to postnatal biventricular correction (modified criteria)

Initial criteria (all of which must be present)	Modified criteria*
LV long-axis Z-score > -2	Aortic stenosis or atresia (mandatory)
LV dysfunction capable of generating ≥ 10 mmHg pressure gradient across aortic valve or ≥ 15 mmHg mitral regurgitation jet gradient	LV long-axis Z-score > -2 (mandatory)
Mitral annulus Z-score > -3	Meet, at least, 4 of the following 5 parameters: • LV long-axis Z-score > 0; • LV short-axis Z-score > 0; • Aortic annulus Z-score > -3,5; • Mitral annulus Z-score > -2; • Aortic valve systolic gradient and/or LV-LA mitral regurgitation ≥ 20 mmHg

LA: left atrium; LV: left ventricle.

* Source: adapted from McElhinney et al.^195^

There is evidence that the transition from normal left ventricle to HLHS in fetuses with critical aortic stenosis almost always occurs during the second or third trimester of gestation.^210^

An interesting aspect observed by the authors is that the progressive growth of left structures during fetal life and early infancy may eventually result in biventricular correction during the first year of life. Applying the strategy initiated with fetal aortic valvuloplasty, treatment continues with neonatal hybrid procedure, which may or may not be associated with a new aortic valvuloplasty or Norwood procedure, with the maintenance of partially restrictive foramen ovale and aortic commissurotomy. This management is a bridge to biventricular correction following the process known as left ventricular rehabilitation.^189,201,211,212^ Although diastolic dysfunction may be a problem in this group of patients, it is believed that this is better than the morbidity and mortality inherent in medium- and long-term of the univentricular pathways.^213^

### 8.4. Critical Aortic Stenosis with Giant Left Atrium

This is a very particular and severe presentation of critical aortic stenosis. In addition to obstructed left ventricular outflow tract, the mitral valve is significantly abnormal, with annular dilation, resulting in severe mitral regurgitation and LA dilation. The foramen ovale is usually quite restrictive, or the interatrial septum is intact, and there is left ventricular endocardial fibroelastosis, which also compromises the subvalvular apparatus of the mitral valve. Most fetuses with this anatomical presentation have some degree of fetal hydrops, with a high risk of death in utero or of triggering premature labor with immediate neonatal death. This disease appears to be the worst spectrum of the mitral valve arcade, where the chordae tendineae are fused and shortened.

It is believed that this anatomical complex primarily compromises the mitral and aortic valves, associated with endocardial fibroelastosis, leading to dilation of left chambers. Restricted left to right flow at the atrial level contributes to significant LA dilation which compresses the right chambers and increases central venous pressure. This seems to be the physiopathology of fetal hydrops, which is present in 70-80% of cases, with polyhydramnios being observed in 100% of cases described by Vogel et al.^44^

Aortic valve opening, in these cases, may reduce the degree of mitral regurgitation and LA pressure, and may treat or improve fetal hydrops and bring the gestation closer to term.^201^ Opening of the atrial septum may be considered for the same procedure, potentializing the effects of aortic valvuloplasty. Besides the intervention, this is a very severe clinical condition, which has a significant impact on fetal and neonatal mortality.

### 8.5. Fetal Pulmonary Valvuloplasty

PAIVS is associated with variable hypoplasia of the right ventricle, tricuspid valve, and right ventricular outflow. The disease's most severe spectrum presents fibromuscular atresia of the infundibulum and pulmonary valve, with significant hypoplasia of the right ventricular cavity and the tricuspid valve, associated with abnormal coronary circulation. Contrastingly, in the more favorable spectrum, the pulmonary valve atresia is membranous; the tricuspid valve annulus diameter and the right ventricular volume are close to normal, and there is an absence of abnormalities in the coronary arteries. Some cases of critical pulmonary stenosis observed during fetal life may evolve to total flow interruption between the right ventricle and the pulmonary artery, with consequent hypoplasia of the right ventricular chamber. These cases behave similarly to PAIVS with mild to moderate hypoplasia of the right ventricle.^214^

The goal of fetal intervention in cases of PAIVS and critical pulmonary stenosis is to promote growth and functional development of the right ventricle and to increase the chances of biventricular circulation during the postnatal period. The identification of potential candidates for the procedure should be based on the risks of the fetus' evolving to univentricular circulation without fetal intervention and the possibility of changing this progression.^198^ The selection of candidates for intervention should follow the criteria previously described in the "Indications" section. Another important criterion in this decision is the presence of signs of fetal heart failure characterized by reverse "a" wave in the ductus venosus flow, which denotes increased right atrium pressure and possible fetal hydrops development. This hemodynamic condition is observed in fetuses who have significant tricuspid regurgitation and very reduced right ventricular compliance.^215^

From the technical point of view, this intervention is more difficult and challenging than aortic valvuloplasty. Due to the reduced dimensions and hypertrophy of the right ventricle, associated with its anatomical characteristics (outflow located anterior and far away from inflow), the positioning of the needle below the pulmonary valve requires very experienced and skilled fetal specialist. The RV puncture should be performed as far as possible from the outflow. In cases with valve atresia, the guidewire utilized should have a slightly firmer tip, in order to allow the interventionist to perforate the valve.^201^ Some authors prefer to introduce a thinner needle through the first one to perforate the valve or proceed the valve perforation with the 17 G needle itself.^200^ After reaching the pulmonary artery, the guide is positioned in one of the pulmonary branches or across the ductus arteriosus, to provide balloon support. For this intervention, the same balloon:annulus ratio as fetal aortic valvuloplasty is employed. The result of the intervention is evaluated by observing the anterograde flow through the pulmonary valve, the reduction of reverse flow through the ductus arteriosus, and the presence of pulmonary insufficiency. Pulmonary insufficiency is a marker of success, and it decreases as gestation advances. Restenosis during fetal life is commonly observed. Most cases will require a new valvuloplasty during the neonatal period.^200,216,217^

In many cases, total recovery of the right ventricle does not occur at birth, making accessory pulmonary flow necessary, either with ductus arteriosus stenting or surgical confection of a systemic to pulmonary shunt (modified Blalock-Taussig).^201^

### 8.6. Fetal Atrial Septostomy

Although HLHS neonatal survival continues to improve worldwide and, slowly in Brazil, some anatomical and functional aspects are risk factors for poor clinical outcome and neonatal or postoperative death.^218^ The presence of an intact atrial septum or severely restricted foramen ovale represents one of the worst risk factors of neonatal mortality. It causes deep hypoxemia after birth and pulmonary hypertension (venocapillary) triggered by pulmonary vein arterialization.^218^

In this condition, resuscitation maneuvers are usually ineffective. Some hospitals recommend emergency Norwood operation, with mortality affecting 83% of patients by the sixth month of life. Even in those who underwent immediate neonatal atrial septostomy, mortality exceeds 48%.^218,219^ These deaths are usually not directly related to the procedure and end up occurring after the first week of life.^196^ It is believed that, in addition to deep neonatal hypoxemia, anatomical abnormalities secondary to in utero venocapillary hypertension are related to mortality. In these cases, anatomopathological studies have demonstrated arterialization of the pulmonary veins associated with lymphatic vessel dilatation.^219-221^ It is estimated that the incidence of severely restrictive foramen ovale or intact interatrial septum associated with HLHS occur in 6% of cases, with some degree of restriction affecting, at least, 22% of patients.^219^

Left atrial decompression during fetal life seems to be essential to the prevent poor immediate neonatal clinical presentation and the remodeling of the pulmonary vascular bed.^196^ The main echocardiographic marker of significantly restricted foramen ovale during fetal life is the presence of high-velocity reverse flow in the pulmonary vein Doppler tracing, which shows an abnormal bidirectional pattern.^45^ This finding indicates that blood is returning to the lungs during atrial contraction, because the LA cannot decompress to the left ventricle or the right atrium.^189^

It is very important to examine at least one pulmonary vein with pulsed-wave Doppler during the echocardiogram of a fetus with HLHS.^222^ The echocardiographer must have in mind that this piece of information may significantly change these patients' outcome and the pre- and postnatal management. Other important features in this condition are pulmonary vein and LA dilation, atrial septum bulging into the right atrium, absent or minimal high-velocity flow across the interatrial septum.^189^

The ideal moment to perform atrial septostomy is discussed.^196,223^ Intending to prevent definitive damage to the pulmonary circulation, the intervention should ideally be performed immediately after the diagnosis. On the other hand, from the technical point of view, it is rather difficult to create an orifice in the interatrial septum that lasts for multiple weeks and prevent severe neonatal hypoxemia. It appears to be consensual that the ideal moment is between the 28^th^ to the 33^rd^ weeks of gestation when the fetus is of good size. During this period, it is feasible to use larger balloons with greater capacity to open wider orifices in the interatrial septum.^190,197^

The use of stents in the interatrial septum has also been considered by some authors.^224,225^ This procedure appears to be more challenging than atrial septostomy, mainly due to the difficulty of optimally positioning the stent in the septum. One of the main problems is to visualize the stent inside the metallic needle via ultrasound. Stent implantation is particularly interesting when the interatrial septum is very thick and, thus, does not allow for the opening of an orifice that is wide enough to alleviate pressure in the LA. Due to the profile of needles available for fetal interventions, the largest stent used is 3 mm, which may, in some cases, reach an internal diameter of 3.5 mm.^39,40^ The rate of poor positioning and embolization is high, according to recent publications. In cases of embolization, the stent is buried in the atrium, without further complications, and the procedure may be completed with the septostomy.^224,225^

### 8.7. Final Considerations of Fetal Cardiac Interventions

With the development of fetal cardiac interventions, several important principles have been recognized. Technical success of the procedure does not always translate to clinical success after birth. Understanding the natural history of the malformation and continuously refining the criteria for patient selection are absolutely critical when one consider the creation of an invasive fetal cardiology program which includes potentially risky procedures. It is important to recognize that the majority of CHD are not fatal, and classic palliative treatment during the neonatal period is an option in many situations. However, for some anomalies whose natural history may be changed for the better, or for those with extremely severe prognoses, fetal intervention may be a therapeutic option. Table 8.3 indicates the class of recommendation and level of evidence for the different fetal cardiac interventions adapted form the Fetal Cardiology guidelines published by the AHA in 2014.^17^

**Table 8.3:** Aim and effects of fetal interventions

Anomaly	Intervention aim	Effect	GOR/LOE
CAS with impending HLHS	Open the Ao valve to promote anterograde flow, stimulate left structure growth, create possibility of biventricular correction	Disease modifying	IIb/B
HLHS with intact IAS or restrictive FO	Open IAS to alleviate left atrial hypertension, prevent pulmonary vasculopathy, improve oxygenation at birth	Lifesaving	IIb/C
CAS with significant mitral regurgitation and giant LA	Open Ao valve and/or IAS, alleviate left atrial hypertension and prevent pulmonary vasculopathy, improve oxygenation at birth	Lifesaving	IIb/C
PAIVS or CPS with evolving RV hypoplasia	Open pulmonary valve to promote right structure growth and lead to possible biventricular repair; treat fetal hydrops in cases of severe tricuspid regurgitation	Disease modifying and/or lifesaving	IIb/C

Ao: aortic; CAS: critical aortic stenosis; CPS: critical pulmonary stenosis; FO: foramen ovale; GOR: grade of recommendation; HLHS: hypoplastic left heart syndrome; IAS: interatrial septum; LA: left atrium; LOE: level of evidence; PAIVS: pulmonary atresia with intact interventricular septum; RV: right ventricle.

Source: Adapted from Donofrio et al.^17^

## Figures and Tables

**Table 5.2:** Group IA. Structural fetal heart diseases without in utero hemodynamic compromise that may progress during fetal life and may or may not require immediate neonatal care. Class of recommendation/level of evidence: IB.^17^,^41^,^57^-^59^

Heart disease	In utero outcome	In utero follow up	Delivery	Postnatal assessment
TOF DORV Complex TGA CTGA TA	May progress to significant obstruction to systemic or pulmonary outflow tracts	After diagnosis, repeat the study every 46 weeks A new study a few weeks before birth is highly recommended	Delivery type according to obstetric indication Level 1; Level 2 or 3 centers in case the in utero hemodynamic condition worsens or precipitates immediate neonatal decompensation (significant obstruction of the systemic or pulmonary outflow tracts)	In all cases, before hospital discharge, cardiac assessment with echocardiogram is required

CTGA: corrected transposition of great arteries; DORV: double outlet right ventricle; TA: tricuspid atresia; TOF: tetralogy of Fallot; TGA: transposition of great arteries.

**Table 5.3:** Group IB. Functional fetal heart diseases without in utero hemodynamic compromise, that not require immediate neonatal care. Class of recommendation/level of evidence: IB.^17^,^41^,^57^-^59^

Heart disease	In utero outcome	In utero follow-up	Delivery	Postnatal assessment
Atrial or ventricular extrasystoles Mild TR	Stable	Repeat the study a few weeks before birth is recommended	Delivery type according to obstetric indication Level 1 center	Maternity ward or outpatient clinic

TR: tricuspid regurgitation.

**Table 5.4:** Group IIA. Structural fetal heart diseases with possible in utero hemodynamic compromise and chance of fetal treatment, which require immediate neonatal care. Class of recommendation/level of evidence: IB.^17^,^41^,^57^-^59^

Heart disease	In utero outcome	In utero follow-up	Delivery	Postnatal assessment
PS PAIVS AS Ebsteins anomaly	Risk of ventricular hypoplasia Risk of ventricular dysfunction or fetal hydrops Risk of circular shunt Risk of fetal arrhythmia	Repeat the study every 2 to 4 weeks is recommended If signs of in utero progression, consider fetal intervention between 22 and 32 weeks If circular shunt, consider induced ductal constriction	Without hydrops, induced vaginal delivery or programmed C-section With hydrops, programmed C-section Level 2 or 3 center	Immediate neonatal cardiac assessment PAIVS requires neonatal treatment Severe or critical PS and AS, may require neonatal treatment Ebsteins anomaly needs treatment if pulmonary atresia and lung hypoplasia

AS: aortic stenosis; PAIVS: pulmonary atresia with intact interventricular septum; PS: pulmonary stenosis.

**Table 5.5:** Group IIA. Structural fetal heart diseases that inevitably require neonatal care. Class of recommendation/level of evidence: IB.17,41,57-59

Heart disease	In utero outcome	In utero follow-up	Delivery	Postnatal assessment
Simple TGA HLHS IAA Severe CoA TAPVR Truncus Complex heart diseases with severely restricted systemic or pulmonary outflow tracts	FO may be restrictive during gestation Although they are complex heart diseases, they tend to remain stable, without hemodynamic compromise during gestation	Repeat study every 4 to 6 weeks is recommended In HLHS or anatomical variations with restrictive ASD, consider fetal intervention Perform a new evaluation a few weeks before delivery	Induced vaginal delivery or programmed C-section Level 2 or 3 center	Immediate neonatal cardiac evaluation The majority are duct dependent CHD and require prostaglandin infusion + interventional or surgical treatment during the first week of life TAPVR and Truncus are diseases with early presentation of HF and PH, and thus require treatment during the first weeks of life, even when they are not duct dependent

CoA: coarctation of the aorta; FO: foramen ovale; HF: heart failure; HLHS: hypoplastic left heart syndrome; IAA: interrupted aortic arch; PH: pulmonary hypertension; TAPVR: total anomalous pulmonary venous return; TGA: transposition of great arteries.

**Table 5.6:** Group IIB. Functional fetal heart diseases with hemodynamic compromise. Class of recommendation/level of evidence: IIb C.17,41,57-59

Heart disease	In utero outcome	In utero follow up	Delivery	Postnatal assessment
Restricted FO Ductal constriction Pericardial effusion Extrinsic compressions Anemia High-output AV fistulas TTTS	May evolve with ventricular dysfunction or fetal hydrops	Serial echocardiogram every 4 to 6 weeks is recommended May need fetal treatment	With hydrops, programmed C-section; Without hydrops, induced vaginal delivery or programmed C-section Level 2 or 3 centers Evaluate the need for preterm delivery	Immediate neonatal cardiac evaluation May require clinical, interventional or surgical treatment immediately after birth

AV: arteriovenous; FO: foramen ovale; TTTS: twin-twin transfusion syndrome.

**Table 5.7:** Group IIB. Nonstructural fetal heart diseases which may evolve with hemodynamic compromise. Class of recommendation/level of evidence: I C.17,41,57-59

Heart disease	In utero outcome	In utero follow up	Delivery	Postnatal assessment
Cardiomyopathies Arrhythmias Tumors	May evolve with fetal hydrops May require medical treatment	Frequent follow-up (weekly or biweekly), depending on diagnosis and hemodynamic compromise	Vaginal delivery in a level 1 center if well controlled tachyarrhythmias or cardiomyopathies without fetal hemodynamic compromise; Programmed C-section in a level 2 or 3 center in cases of arrhythmia or hydrops which have not been resolved in utero	Cardiac management according to diagnosis Treatment is usually with medication, with the exception of some tumors which need to be removed due to obstructive or compressive character, which compromises hemodynamics

**Table 7.2:** In utero management of bradycardias

Diagnosis	Primary causes	In utero management	GOR/LOE	Comments
Sinus bradycardia	Ectopic atrial pacemaker	Rule out fetal distress as the cause of bradycardia	I/A	Can be seen in atrial isomerism
	Sinus node dysfunction (including immune mediated or infection)	Observation until bradycardia resolves	I/A	Test for anti-Ro/LA antibodies Maternal IgG/IgM for TORCH diseases and parvovirus
	Secondary causes: maternal medications, maternal hypothyroidism, fetal distress or fetal CNS abnormalities	Treat underlying cause of bradycardia	I/A	
Blocked atrial bigeminy	Atrial extrasystoles	Observe / reduce maternal stimulants	I/A	10% risk of fetal SVT Weekly auscultation of fetal HR until arrhythmia resolves
AVB	Maternal anti-Ro/La antibodies	Observation	I/A	Structurally normal heart
		Dexamethasone for second-degree block or first-degree block with findings of cardiac inflammation	IIb/B	Endocardial fibroelastosis, associated valvular or myocardial dysfunctions
		For CAVB to prevent death or cardiomyopathy	IIb/B	4-8 mg/day
		IVIG (note: IVIG as prophylaxis is not recommended)	IIa/C	
		Sympathomimetics for HR < 55 bpm or higher rates associated with fetal hydrops	Ib/C	
	CAVB not related to antibodies	Observation	I/A	Associated with structural defects such as CTGA, left atrial isomerism
	CAVB related to channelopathies	Observation	I/A	
		Avoid QT-prolonging drugs		

AVB: atrioventricular block; CAVB: complete atrioventricular block; CNS: central nervous system; CTGA: corrected transposition of great arteries; GOR: grade of recommendation; HR: heart rate; IVIG: intravenous infusion of gammaglobulin; LOE: level of evidence; mg: milligrams; SVT: supraventricular tachycardia; TORCH: toxoplasma IgG, Rubella IgG, Cytomegalovirus IgG, and Herpes. Source: adapted from Donofrio et al.^17^
